# Size-Controlled Mesoporous Silica Nanoparticles via Template Nanoarchitectonics from a Deferoxamine Derivative for Enhanced Blood–Brain Barrier Permeability and Neuroprotective Chelation Therapy

**DOI:** 10.1021/acsami.5c18528

**Published:** 2025-12-11

**Authors:** Mónica Onrubia-Márquez, Francisco Navas, Esther M. Sánchez-Carnerero, Antonio Martín, Anselma Liturri, Morena Miciaccia, Raúl Sanz, Antonio Scilimati, Rafael A. García-Muñoz, Maria Grazia Perrone, Victoria Morales

**Affiliations:** † Department of Chemical and Environmental Technology, 16776 ESCET, Universidad Rey Juan Carlos, 28933 Móstoles, Madrid, Spain; ‡ Instituto de Investigación de Tecnologías para la Sostenibilidad, Universidad Rey Juan Carlos (URJC), 28933 Móstoles, Madrid, Spain; § Research Laboratory for Woman and Child Health, Department of Pharmacy-Pharmaceutical Sciences, 9295 University of Bari “Aldo Moro”, 70125 Bari, Italy

**Keywords:** Deferoxamine, neuroprotection, chelation therapy, mesoporous silica nanoparticles, blood−brain barrier, amyloid misfolding

## Abstract

Neurodegenerative diseases (NDs) are progressive and fatal disorders that primarily affect the elderly and remain incurable. Characterized by irreversible neuronal loss, they leave patients increasingly dependent on caregivers. Despite diverse clinical presentations, NDs share common pathological features, such as protein aggregation, metal accumulation, oxidative stress, and chronic neuroinflammation. Despite numerous efforts, most therapeutic candidates fail due to poor efficacy, toxicity concerns, or limited blood–brain barrier (BBB) permeability, thereby highlighting the need for enhanced formulations. Nanomedicine offers a promising strategy to improve the therapeutic performance of existing compounds. This study presents a nanoformulation of the metal chelator deferoxamine (DFO) based on the drug-structure-directing agent (DSDA) concept, in which a hydrophobic chain is covalently linked to the DFO molecule to impart amphiphilic properties and acts as a template for the synthesis of a mesoporous silica nanoparticle (MSN). This approach allows for the one-pot fabrication of DFO-loaded MSNs (DFO@MSNs) with controlled sizes of below 20 nm without the need for surfactant removal. Compared to MCM-41-based systems, DFO@MSNs exhibited a higher drug loading capacity (10 mg of DFO/100 mg of MSNs) and a significantly more sustained release profile, minimizing premature leakage, with less than 20% of the cargo released over 24 h. Safety of DFO@MSN was assessed using BV-2 microglial and human neuroblastoma SH-SY5Y cell lines, and *in vitro* assays confirmed its enhanced iron-chelating capacity and effective inhibition of aluminum-induced amyloid aggregation. Furthermore, permeability studies using a Caco-2 *in vitro* BBB model revealed that a smaller particle size greatly enhances transport across the barrier. These results support DFO@MSNs as a promising multifunctional nanoplatform for targeted chelation therapy and neuroprotection in ND treatment.

## Graphical abstract

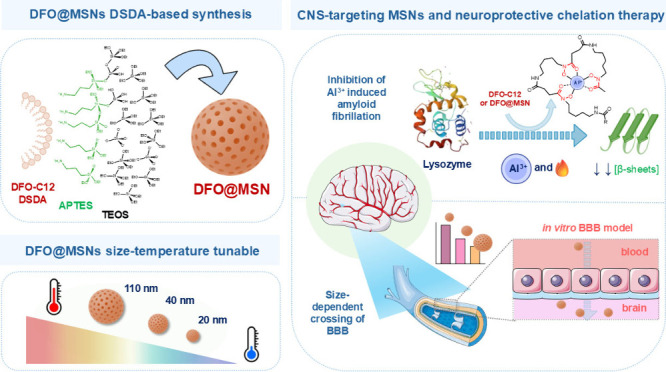

## 1 Introduction

Neurodegenerative diseases (NDs) are fatal and incurable disorders that severely impair the ability of those affected to perform daily activities. While the symptoms vary from one disease to another, they all lead to irreversible brain cell degeneration. The most common of these are Alzheimer’s disease (AD) and Parkinson’s disease (PD).^1^ AD is a form of dementia characterized by cognitive decline, affecting memory and communication skills,^2^ meanwhile, PD is marked by the presence of bradykinesia and other symptoms affecting not only but mainly the motor system.^3^ Brain disorders accounted for more than 15% of worldwide health loss, making them one of the leading causes of health decline.^4^ The diagnosis of these diseases is devastating not only for the patients, but also for their caregivers, directly impacting their careers, health or socioeconomic status.^5^ Global healthcare spending in dementia increases every year and is estimated to triple by 2050.^6^ Agencies, such as the World Health Organization (WHO), call for an urgent public response to slow the rising incidence of ND and prevent socioeconomic costs from overwhelming healthcare system.

The causes of neuronal degeneration remain unknown, but they all share a common pathophysiology. Some of the hallmarks common to various neurodegenerative diseases include aberrant protein aggregation, abnormal metal accumulation, chronic inflammation, or neurotoxicity due to reactive oxygen species (ROS) production. These processes appear to be interconnected, leading to neuronal cell death.^7^ Therefore, multitargeted therapies addressing multiple pathological mechanisms are essential. New compounds or nanoparticles combining therapeutic strategies have shown efficacy in mitigating several key hallmarks of neurodegeneration, from preventing protein misfolding to mitigating mitochondrial oxidative stress, demonstrating the potential of multitargeting approaches for treating these diseases and addressing the complexity of neurodegeneration process.^8,9^

Dysregulation of metal homeostasis in the brain and abnormal accumulation of metals are present in different neurodegenerative diseases.^10,11^ Senile plaques, a characteristic pathological feature of AD, is linked with abnormal metal accumulation.^11,12^ These plaques are formed by the amyloid beta peptide (Aβ), which consists of 40–42 amino acids and is the result of the enzymatic cleavage of the amyloid precursor protein, a transmembrane protein that is widely expressed in the central nervous system (CNS). Aberrant aggregation of Aβ forms insoluble oligomers, which then form fibrils that finally result in amyloid plaques.^7,13^ The presence of Al­(III) and Fe­(III) in the brain can promote the formation of these oligomers and their pathological deposition.^14,15^ These protein aggregates are toxic and trigger immune responses that lead to chronic inflammation or, in some cases, impair clearance pathways.^16^ Additionally, Fe­(III) binding to Aβ induces a redox reaction that catalytically generates H_2_O_2_, thereby increasing ROS levels in the surrounding environment.^17^ Unlike iron, aluminum is not an essential metal; however, in its trivalent form, it is highly reactive, binding to proteins and accumulating into several tissues. Aluminum is particularly toxic to the CNS, and elevated levels of aluminum have been detected in Aβ plaques in the brain tissue of AD patients, suggesting a link to neurodegeneration.^10^ In PD, protein misfolding is also observed, consisting of abnormal aggregates of α-synuclein (α-syn). *In vitro* studies have shown that the presence of metals catalyzes the formation of α-syn fibrils. In fact, there is a clear relationship between elevated concentrations of iron in the Sustantia Nigra of PD patients with a mitochondrial dysfunction, elevated concentrations of ROS and high levels of misfolded α-syn.^18,19^ Taking all of the above into account, abnormal protein aggregation, excessive ROS production, and neuroinflammation appear to be part of a vicious cycle contributing to neurodegeneration in AD and PD.

Compelling scientific evidence implicating metals in the pathogenesis of NDs has led to the investigation of metal chelators as promising treatments for these disorders. Deferoxamine (DFO), a drug approved by Food and Drug Administration (FDA) and European Medical Agency (EMA), is a hexadentate metal chelator with high iron affinity (log β = 41.6).^20^ It is typically used to treat iron overload in patients who receive frequent blood transfusions. As many drug candidates fail in clinical trials, repurposing strategies have been explored. Indeed, the iron chelator DFO has been evaluated in Alzheimer’s trial, showing reduced decline in daily living skills in the group treated with DFO.^21^ Despite these promising outcomes, DFO treatment presents several challenges. DFO exhibits a notably short plasma half-life of 20–30 min, requiring high doses to be administered via subcutaneous injection for 5–7 days to ensure efficacy. Additionally, high doses of DFO are associated with nonspecific toxicities.^22^ To overcome these limitations and reach CNS, new formulations are being developed to allow for sustained and prolonged release of DFO.^22–24^ In addressing this issue, Ul-Haq et al. synthesized a dendrimer that increased the circulation lifetime of DFO by 484-fold. The effectiveness of the nanochelator is evident from the increased levels of iron excreted by the groups of animals that were treated. Additionally, the synthesized dendrimer increased the LD_50_ from 250 mg·kg^–1^ to 1000 mg·kg^–1^.^25^ In a similar report, DFO conjugated with hyperbranched polyglycerol showed good results when it was administered to animal models. Mice exposed to DFO conjugate did not show signs of toxicity, maintaining their body weight and showing normal levels of lactate dehydrogenase in serum.^26^ Another study showed that the DFO-based nanochelator was effective in treating iron overload. It demonstrated rapid renal clearance and avoided the toxicities typically associated with long-term, high-dose DFO treatment.^27^ Moreover, DFO engineered materials have also been developed for the treatment of ND as well. You et al. tested their polymeric DFO nanoparticles in a PD mouse model. These nanoparticles enhanced the performance of the treated group in the rotarod assay. The nanoformulation was able to cross the blood-brain barrier (BBB) and effectively remove iron from the brain without any damage to the tissue.^28^ In a recent report, black phosphorus nanosheets containing polydopamine and DFO showed excellent results by reducing dopaminergic neuron loss, regulating brain iron levels and inhibiting ferroptosis.^29^ Additionally, these nanomaterials demonstrated the ability to cross the BBB *in vitro* due to their small size and a specialized functionalization that facilitated their transport.

BBB restricts the passage of exogenous compounds to the brain, thereby limiting drug efficacy. Many drug candidates fail clinical trials due to poor efficacy or pharmacokinetics. The outlook is even worse for CNS drugs, as many candidates fail due to low BBB penetration.^30^ The rise of nanomedicine has introduced new strategies to enhance BBB crossing, with the nanoparticle size being a key factor. Although there is no consensus, it is generally considered that nanoparticles larger than 100 nm are unable to cross BBB.^31^ An *in vitro* permeability study found that 30 nm nanoparticles traversed the cell monolayer model simulating the BBB, three times more effectively than 100 nm ones.^32^ Similarly, a study evaluating nanoparticles with sizes of 25, 50, and 100 nm revealed a size-dependent penetration mechanism.^33^ These findings confirm that size optimization is crucial in nanoparticle design.

Mesoporous silica nanoparticles (MSNs) have attracted significant attention as drug delivery systems (DDSs) due to their unique properties. These materials offer a high surface area (some up to 1000 m^2^·g^–1^) and a pore diameter in the range of 2–50 nm, facilitating effective drug loading.^34,35^ Additionally, the synthesis of MSNs is highly versatile, allowing their sizes to be adapted for specific applications. MSNs also exhibit remarkable biocompatibility and stability, minimizing toxicity issues and extending their circulation time within biological systems.^35,36^ The most common process for drug encapsulation in MSNs is adsorption method.^37^ For successful drug adsorption onto the silica surface, favorable interactions between the host and the drug candidate are crucial. Although MSNs can have their surface charge adjusted, weak interactions between the drug and the MSN may lead to premature drug release. To the best of our knowledge, only two studies have explored the use of MSNs in chelation therapy. In one case, the chelator quercetin was encapsulated in 260–280 nm-sized MSNs,^38^ while Yang et al. synthesized Au-MSNs as small as 68 nm for clioquinol loading through adsorption methods.9 However, both systems exhibited a burst drug release profile with a plateau within 24 h due to the weak drug-matrix interactions. Therefore, given the importance of long-term dosing in DFO-based therapies, the design of nanomaterials capable of providing sustained drug release remains a major challenge.

In our research group, a new strategy has been developed to synthesize MSNs using an amphiphilic derivative of the drug as a structure-directing agent (DSDA). Due to their amphiphilic nature, DSDAs behave as surfactant in aqueous solution, forming micelles above its critical micelle concentration (CMC) with the hydrophobic chains packed inside and the polar head exposed to the aqueous phase. This innovative approach avoids the needs to remove the surfactant after the MSN synthesis, as the template has inherent pharmacological activity.^39^ Therefore, drug loading using the DSDA approach is maximized. Additionally, *in vitro* release assays conducted at various physiological pH levels have demonstrated the potential for controlled, sustained drug release using this new strategy.^40,41^

Here, we present a DFO formulation based on DSDA-MSN synthesis that demonstrates therapeutic potential in mitigating features related to neurodegenerative disorders. The properties of MSNs templated with a DFO derivative were optimized by investigating the effects of aging time, DSDA concentration, and temperature of the synthesis. Temperature variation significantly influences the size control in MSNs, enabling the production of nanoparticles smaller than 20 nm. DFO@MSN synthesized from DSDA exhibits a continuous, sustained drug release profile, preventing the premature release observed for DFO conventionally adsorbed, in a MCM-41, used as refence material. *In vitro* studies revealed that DFO@MSN has a higher iron-chelating capacity than that of the reference material. Additionally, these nanoparticles inhibit amyloid fibril formation in the presence of aluminum, rendering DFO@MSN a promising multitarget therapy for neurodegenerative diseases. Finally, we demonstrate that DFO@MSNs cross the BBB in a size-dependent manner, confirming their suitability as chelation therapy with potential applications in the treatment of ND (Figure 1).

**Figure 1:**
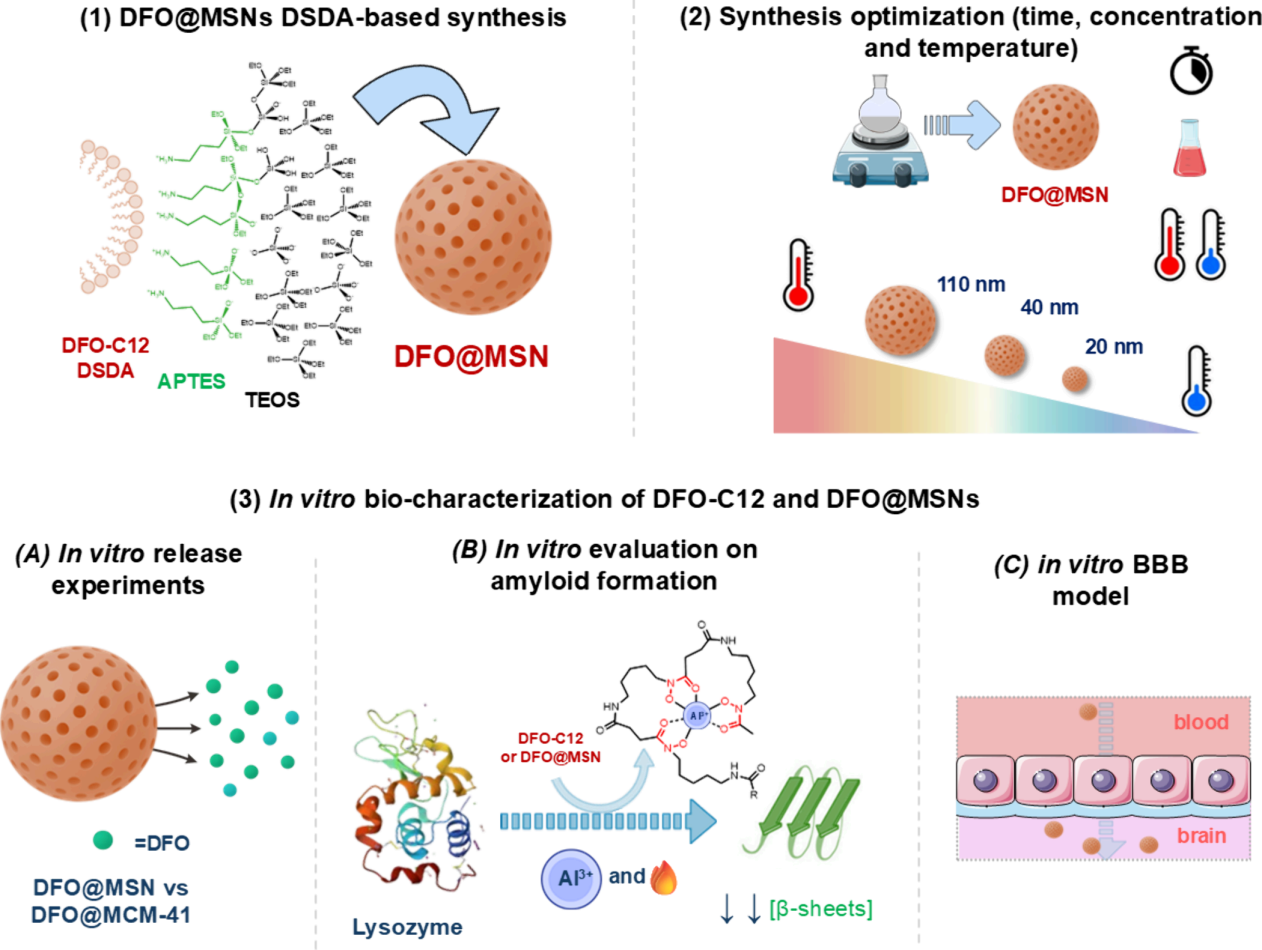
Schematic overview of the work. (1) DFO@MSN DSDA-based synthesis, showing the coassembly of DSDA DFO-C12, APTES, and TEOS leading to DFO@MSN. (2) Synthesis optimization, including the effect of time, concentration, and temperature on particle size. (3) *In vitro* bio-characterization of DFO-C12 and DFO@MSNs, including (A) *in vitro* release experiments comparing DFO@MSN with DFO@MCM-41, (B) *in vitro* evaluation of amyloid formation using lysozyme,and (C) *in vitro* BBB permeability model.

## 2 Materials and Methods

### 2.1 Chemicals

Deferoxamine mesylate (DFO, 99%) was purchased from Apollo Scientific. 3-(Dodec-2–1-yl)­dihydrofuran-2,5-dione (97%), N,N-diisopropylethylamine (DIPEA, 99%), (3-aminopropyl) triethoxysilane (APTES, 98%), tetraethylorthosilicate (TEOS, 98%), cetyltrimethylammonium bromide (99%), (3-aminopropyl)­trimethoxysilane (APTMS, 97%), (3-cyanopropyl)­triethoxysilane (CPTES, 97%), octanol (99%), iron­(III) chloride (FeCl_3_, 99%), iron­(III) chloride hexahydrate, aluminum­(III) chloride hexahydrate, dimethyl sulfoxide-D6 (99.8% D), cell counting kit-8 (CCK-8), 3-(4,5-dimethyl-2-thiazolyl)-2,5-diphenyl-2H-tetrazolium bromide (MTT), ammonium molybdate (99%), fluorescein isothiocyanate isomer I (FITC, >90%), hexadecyltrimethylammonium bromide (CTAB, >96%), and ThioflavinT (ThT) were obtained from Sigma-Aldrich. Anhydrous dimethylformamide (DMF, 99%) was purchased from Carlo Erbo. Hank’s balanced salt solution (HBSS) and phosphate buffered saline (PBS) were obtained from Corning. Ethanol, hydrochloric acid (HCl, 37%), sulfuric acid (98%), and dimethyl sulfoxide (DMSO, 99%) were purchased from QUIMIPUR. Chicken egg white lysozyme (CEWL) was obtained from Molekula. Cell mediums were purchased from Invitrogen.

### 2.2 Synthesis of DSDA DFO-C12

DSDA DFO-C12 was synthesized by amidation reaction of the free amino group of DFO with the anhydride group of 3-(dodec-2-1-yl)­dihydrofuran-2,5-dione (Figure S1). The reaction was performed as follows: in a round-bottom flask equipped with a magnetic stirrer, 3-(dodec-2-1-yl)­dihydrofuran-2,5-dione (101 mg, 0.38 mmol, 1 equiv), DFO (250 mg, 0.38 mmol, 1 equiv), and DMF anhydrous (5 mL) were added under inert atmosphere and heated at 60 °C. The mixture turned colorless upon complete dissolution, and DIPEA (100 μL, 0.57 mmol, 1.5 equiv) was added dropwise. The mixture was stirred for 24 h under an inert atmosphere at 60 °C. Afterward, the solvent was removed under reduced pressure. The solid was repeatedly washed with water to remove the excess base and unreacted anhydride. DSDA DFO-C12 was obtained as a white solid (255 mg, 81% yield).

### 2.3 Synthesis of DFO@MSNs

The synthesis of MSNs using DSDA-DFO-C12 as template was performed according previous reported protocol for the synthesis of MSNs using anionic surfactant with minor modifications.^39^ For this, DSDA DFO-C12 (180 mg, 0.22 mmol, 1 equiv) was dispersed in basic aqueous media (pH = 9). The solution was heated until complete dissolution (colorless solution). Later, APTES (94 μL, 0.402 mmol, 1.85 equiv) was added under stirring. After a few minutes, the reaction mixture turned white. At that moment, TEOS (430 μL, 1.92 mmol, 8.85 equiv) was added dropwise. The reaction mixture was stirred at 75 °C for 24 h. To afford a precipitation of the gel formed, the reaction mixture was aged at 100 °C. This solid was filtered and washed repeatedly with water. Aging time was extended from 24 to 72 h. The influence of DSDA concentration was investigated, reducing it from 26 to 13 mM. Temperature of reaction was investigated varying it from 65 to 85 °C. The synthetic conditions employed among yield of reactions are shown in Table 1.

**Table 1:** Synthetic Conditions of DFO@MSNs

Nanomaterial	DSDA:APTES:TEOS:H_2_O	Synthesis temperature/time
DFO@MSN-1/DFO@MSN-40	1:1.85:8.85:2135	75 °C 24 h, then 100 °C 24 h
DFO@MSN-2	1:1.85:8.85:2135	75 °C 24 h, then 100 °C 72 h
DFO@MSN-3	0.5:1.85:8.85:2135	75 °C 24 h, then 100 °C 24 h
DFO@MSN-20	1:1.85:8.85:2135	65 °C 24 h, then 100 °C 24 h
DFO@MSN-110	1:1.85:8.85:2135	85 °C 24 h, then 100 °C 24 h

### 2.4 Synthesis and Functionalization of MCM-41 Sample Used As Reference Material

In a typical synthesis of MCM-41, as reference material,^42^ CTAB (160 mg, 0.44 mmol, 1 equiv) was added to a mixture of milli-Q water (68 mL) and ethanol (10 mL). Then, the pH of the solution was adjusted to 12 using 1 M NaOH (820 μL). The reaction mixture was heated to 82 °C under magnetic stirring until a clear solution is obtained. TEOS (820 μL, 3.94 mmol, 9 equiv) was added dropwise. The reaction was stopped after 2 h on ice. The solid was collected by filtration, washed with milli-Q water and ethanol, and dried under vacuum. The surfactant was removed by calcinating the sample at 550 °C for 5 h, with ramp of 1.8 °C·min^–1^.

The functionalization of the reference MSNs with an amine group (MCM-41-NH_2_) was performed as follows. The first step consisted of rehydroxylation of calcinated silica samples. For this, 100 mg of MCM-41 were dispersed in 15 mL of HCl (18.5%) and stirred at 70 °C during 12 h. The material was recovered by filtration and washed with distilled water and ethanol. The solid was dried under vacuum overnight. In step two, 100 mg of these MCM-41 were dispersed in 40 mL of anhydrous toluene. The mixture was heated to 110 °C. Then, an excess APTMS (2 mL) was added. The reaction was carried out for 24 h under an argon atmosphere. The precipitate was filtered, washed with toluene and ethanol and dried under vacuum.

The functionalization of the MCM-41 with carboxylic acid groups (MCM-41-COOH) was performed using a published protocol.^41^ For this, 100 mg of calcinated MCM-41 were dispersed in 25 mL of anhydrous toluene and CPTES (1.25 mL) were added. The reaction was carried out at 80 °C for 24 h. The oxidation of nitrile groups was performed using 48% sulfuric acid at a rate of 12 mL/100 mg of MSNs. The solid was filtered and washed with water and ethanol.

### 2.5 DFO Loading Procedure in Reference Material (MCM-41)

The adsorption of DFO on MCM-41-NH_2_ and MCM-41-COOH was performed according previous report in literature.^43^ 50 mg of reference material was dispersed in 5 mL of milli-Q water. Once the material is homogeneously dispersed, an amount of DFO was added to reach final concentrations of 7 and 15 mg·mL^–1^ of DFO. The mixture was stirred for 24 h at 37 °C. Then, the DFO@MSN-COOH or DFO@MSN-NH_2_ was recovered by filtration, and the excess DFO was removed by washing with 5 mL of distilled water. The amount of DFO loaded was quantified by thermogravimetric analysis (TGA).

### 2.6 Characterization

^1^H and ^13^C NMR spectra were recorded on a Varian Infinity 400 MHz spectrometer fitted with a 9.4 T magnetic field. Chemical shifts are reported in parts per million (δ ppm) and they were externally referenced to tetramethyl silane. Mass measurements were performed on an ultrahigh-performance liquid chromatography tandem mass spectrometry (UHPLC-HESI-MS/MS) using VIP heated electrospray ionization interface (Bruker UHPLC/MSMS EVOQ ELITE) with a triple-quadrupole detector. Surface tension (γ) measurements as function of surfactant concentration, were performed using a surface force tensiometer Krüss K10 with a Pt Wilhelmy plate as a probe to determine the critical micelle concentration (CMC) of DFO-C12. Nitrogen adsorption measurements were performed on a Micromeritics TriStar 3000 instrument. The calcined samples were degassed prior to measurement. In this process, 100 mg of calcinated MSNs were heated to 100 °C for 24 h in vacuum. Nitrogen (N_2_) adsorption–desorption measurements were performed at 77 K. Pore size was calculated by an NLDFT model on the adsorption branch of the isotherms. The elemental analysis was performed using a CHNS-O analyzer Flash 2000 Thermo Scientific apparatus. Fourier transform infrared spectroscopy equipped with attenuated total reflectance accessory (FTIR-ATR) spectra were recorded in the wavenumber range from 4000–400 cm^–1^ with an FTIR Nicolet iS50 (ThermoFisher Scientific) spectrometer with ATR. A resolution of 8 cm^–1^ and 32 scans/sample was used. The morphologies and sizes of MSNs were investigated with a transmission electron microscopy (TEM) JEOL JEM 1400 operated at 200 kV with a resolution of 0.25 nm. An X-ray detector (EDS, energy dispersive spectroscopy system model TEM XPlore from Oxford Instruments) was used for elemental mapping. ImageJ software was used to determine the diameters of nanoparticles in TEM images. The diameter of nanoparticle (D_N_) is expressed as the mean ± SD resulted from measuring 20 random nanoparticles. Previously, the samples were dispersed in ethanol and deposited on a carbon-coated copper grid. Dynamic Light Scattering (DLS) measurements were performed on a Nanoplus DLS/Zeta Potential from Particulate Systems. Calcinated samples were suspended at a concentration of 0.25 mg·mL^–1^ in ethanol. Z-potential of DFO@MSNs was measured in PBS in triplicate and expressed as mean ± SD.

Thermogravimetric analyses (TGA) were performed under an air atmosphere with a Star system Mettler Thermobalance in the temperature range from 40 to 800 °C at 5 °C min^–1^. The organic content (wt%) was calculated from the weight loss of samples which were dried in at oven at 40 °C overnight. The% of DFO loaded by DSDA method was calculated assuming a complete incorporation of APTES. Loading capacity (LC) and encapsulation efficiency were determined using the formulas: (LC%)=(weight of DFO incorporated/weight of DFO@MSN) × 100 and (EE%) = weight of DFO incorporated/weight of DFO added during the synthesis) × 100.

### 2.7 Determination of Log P Values

The octanol–water partition coefficients (log P) were determined using the shake flask method.^44^ Briefly, milli-Q water (25 mL) and octanol (25 mL), in equal ratio, were stirred together for 72 h to allow saturation of both phases. A solution of 10^–3^ M DSDA DFO-C12 was prepared first in water, and then the same volume of octanol was added. Biphasic solution was shaken for 10 min, and the concentration of the DSDA and DFO in both phases was determined by RP-HPLC-MS on a reversal phase high performance liquid chromatography (Bruker UHPL/MSMS EVOQ QUBE): flow rate, 1 mL/min; gradient solvent system H_2_O:methanol (A/B) and formic acid at 0.05%, initial 98%A + 2% B; 10 min linear gradient to 100% B. Reported log is defined as log­[compound]_oct_/[compound]_water_.

### 2.8 Chelation Assay of DFO-C12 DSDA and DFO@MSN

The formation of an Fe­(III) complex with DFO-C12 DSDA was investigated by a method of continuous variations. Solutions of FeCl_3_ and DFO-C12 DSDA were prepared in a mixture H_2_O:DMSO (9:1). Several absorbance measurements varying mole fractions of metal–ligand were recorded by UV–vis at 430 nm (Evolution 201 UV–vis spectrophotometer, ThermoScientific). The maximum of the plot corresponds to binding stoichiometry of Fe­(III) and the synthesized compound, DFO-C12 DSDA.

In case of MSNs the assay was adapted from previous published protocol.^45^ The chelation capacity of nanoparticles was investigated dispersing 6 mg of DFO@MSN into FeCl_3_ solution (in excess) at 37.5 °C for 6 and 24 h. The samples were centrifuged and washed with water. The solid was examined by EDS mapping. Then, nanoparticles were digested microwave assisted according to previous reported protocol,^46^ in a mixture of HNO_3_:HF (9:1). After digestion, 0.3 equiv of H_3_BO_3_ were added. The solution was analyzed by ICP-OES to quantify iron present in DFO@MSN after chelation assay. The ICP-OES analysis was performed in an ICP-OES Agilent 5800 VDV mass spectrometer using axial configuration and an aqueous matrix. The calibration of the device used was from 189 to 789 nm, and the resolution was 1 pm.

### 2.9 *In Vitro* Release Experiments

The drug release protocol was adapted from previous published protocol for DFO.^47^ The kinetics of the release of DFO were studied as follows: a solution of 6 mg·mL^–1^ of nanoparticles containing DFO was incubated at 37.5 °C in a thermoshaker with vigorous shaking (900 rpm). To quantify DFO released to medium, solution was centrifuged, and an excess of Fe­(III) was added to the supernatant. The formation of the [DFO-Fe]^+^ complex (with stoichiometry 1:1) was confirmed by the appearance of an absorption band at 430 nm. This mixture was analyzed by a UV–vis spectrometer (Evolution 201 UV–vis Spectrophotometer, ThermoScientific) at 430 nm. After each measure, a fresh medium was added. *In vitro* release experiments were performed in PBS (pH = 7.4). The measurements are expressed as the mean ± SD from three independent experiments.

### 2.10 Cell Cultures

All cell lines were purchased from the American Type Culture Collection (ATCC). Caco-2 (human colon adenocarcinoma cell line) and BV-2 (mouse microglial cell line) were grown in Dulbecco’s Modified Eagle’s Medium high glucose (DMEM) supplemented with 10% fetal bovine serum (FBS), 2 mM glutamine, 100 μg·mL^–1^ penicillin, and 100 μg·mL^–1^ streptomycin. Cell cultures were maintained at 37 °C in a humidified atmosphere with 5% CO_2_. Human neuroblastoma SH-SY5Y cells were cultured at 37 °C in 5% CO_2_ as in DMEM/F-12 with an l-glutamine medium composed of 10% FBS, 1% penicillin/streptomycin.

### 2.11 Cell Viability Assay

Briefly, 10.000 BV-2 cells/well in 100 μL of culture medium were seeded into 96-well plates (transparent plate, clear bottom; Costar, Corning Inc.) and incubated overnight at 37 °C in 5% CO_2_. Stock solutions of the drug were preparate in DMSO, keeping it concentration <1% in the experiments. Different concentrations of DFO, DFO-C12 and MSNs dispersions (25–100 μM) in 100 μL of medium were added to the cells. Stock solutions were prepared just before their use. Control wells with DMSO were used to evaluate the possible cytotoxicity of the solvent. After 72 h incubation, CCK-8 (10 μL) was added to each well, and after 3–4 h of incubation at 37 °C, the absorbance values at λ = 450 nm were determined on the Tecan Infinite 200 microplate reader. The cytotoxicity assay for SH-SY5Y was conducted by seeding cells at a density of 40.000 cells/well for 24 h in 96-well plate and incubated overnight. Subsequently, the culture medium was replaced with 100 μL of fresh medium containing dilutions of drug and MSNs ranging from 10 to 100 μM of DFO. The plate was incubated for 24 h. After this time, MTT (10 μL) was added to each well, and after 3–4 h of incubation at 37 °C, the absorbance values at λ = 540 nm were determined on the Tecan Infinite 200 microplate reader.

Data were plotted as % cell viability, normalized vs untreated cells by using GraphPad Prism software version 7.05. Values are expressed as the mean ± SEM of at least three independent experiments. Two-way ANOVA (treatment x concentration), followed by Tukey’s multiple comparisons test was used to analyze the difference between the means of treatments and concentrations;^48^ *p* < 0.05 was considered significant.

### 2.12 Permeability experiment

#### 2.12.1 Preparation of Caco-2 Monolayer

The preparation of the Caco-2 cell monolayer as an *in vitro* BBB model was performed as follows: Caco-2 cells were seeded at a density of 10.000 cells per well on a Millicell 96-well cell culture plate. The culture medium was replaced every 48 h. The formation of the monolayer was monitored by measuring the transepithelial electrical resistance (TEER) using an epithelial voltohmmeter (Millicell-ERS). After 21 days of culture, the Caco-2 cell monolayers were used for permeability experiments. All TEER values measured immediately before the experiments were above 300 Ω·cm^2^, confirming the integrity of the monolayer.^49^

#### 2.12.2 Drug Transport Experiment

Drug transport experiments were carried out following the previously published protocol.^50^ Briefly, after 21 days of culture, the medium was removed, and the cells were washed twice with Hank’s Balanced Salt Solution (HBSS) to eliminate any residual culture medium. Subsequently, the wells were refilled with HBSS and incubated at 37 °C for 30 min. After incubation, the HBSS was removed, and solutions containing the compound were added either to the filter well (apical side) or to the receiver plate (basolateral side), while HBSS without the compound was added to the corresponding opposite side. The final concentration of the compounds was 10^–4^ M. The plates were then incubated at 37 °C for 2 h. At the end of the incubation period, the samples from both compartments were collected and analyzed by UV–vis spectroscopy. The apparent permeability coefficient (Papp), expressed in nm·s^–1^, was calculated using the following equation:

1 $$\mathrm{Papp}=\left[\mathrm{VA}/\left(\mathrm{area}\times \mathrm{time}\right)\right]\times \left({\left[\mathrm{drug}\right]}_{\mathrm{acceptor}}/{\left[\mathrm{drug}\right]}_{\mathrm{receptor}}\right)$$

where VA is the volume (mL) in the acceptor well, area is the surface area of the membrane (0.11 cm^2^ of the well), time is the total transport time (7200 s), and [drug] is drug concentration measured by UV–vis spectroscopy.

#### 2.12.3 Nanoparticle Preparation and Permeability Assay

To evaluate the transport of DFO@MSNs across the Caco-2 monolayer, a modified drug transport experiment was performed. First, for the preparation, calcinated samples of DFO@MSNs were rehydroxylated as follows: 50 mg of the calcinated sample of DFO@MSN was stirred overnight in a 1:1 mixture of HCl:H_2_O at 70 °C. The nanoparticles were then recovered by filtration. The recovered solid was subsequently dispersed in toluene, and an excess of APTMS was added to the solution to incorporate the amino groups onto the particle surface. After 24 h, the solid was filtered and dried under vacuum. Finally, 10 mg of DFO@MSNs functionalized with amino groups were dispersed in 2.5 mL of solvent, and a 760 μM solution of FITC in ethanol was added. The resulting product was filtered and stored in the absence of light at 17 °C. To perform permeability experiment, a concentration of 50 μg·mL^–1^ of DFO@MSN-FITC was used following the same protocol described for drug permeability determination. The concentration of nanoparticles on both sides of the membrane was determined by fluorescence spectroscopy, according to a previously published protocol.^51^ Fluorescence measurements were performed by using a Tecan Infinite 200 plate reader.

### 2.13 Amyloid Fibrillation Assay

#### 2.13.1 Preparation of Lysozyme and Solutions

Lysozyme (Lys) was dissolved in Milli-Q water and then dialyzed overnight against pure water at 4 °C. Dialyzed protein was lyophilized and stored at – 20 °C. To prepare the different assays,^52,53^ the crystalline powder lyophilized was resuspended in solution of HCl at pH 1.6, containing 200 ppm of NaN_3_ to prevent bacterial growth. The concentration of protein was determined measuring absorbance at 280 nm in UV–vis spectrometer (Evolution 201 UV–vis spectrophotometer, ThermoScientic) and using reported extinction coefficient ε = 2.64 mL·mg^–1^·cm^–1^.^54^ A final concentration of lysozyme of 200 μM was used in the protein aggregation studies. To evaluate the effect of metals and chelators in aggregation process, protein solutions were mixed with XCl_3_·6H_2_O (X = Fe and Al) and DFO treatments (DSDA DFO-C12 and DFO@MSN) in 1:1:1 molar ratio. MCM-41 was used as the reference material. Solutions were incubated at 65 °C at a thermoshaker working at 500 rpm.

#### 2.13.2 ThT Fluorescence Assay

CEWL fibrillation in the presence of metals and chelators were analyzed by fluorescence spectroscopy (FL 6500 Fluorescence Spectrophotometer, PerkinElmer). Aliquots of experiment were diluted in PBS containing 13.3 μM ThT. To measure ThT fluorescence intensity, 440 nm was used as the excitation wavelength with a slit width of 5 nm. The emission fluorescence spectra was recorded from 460 to 550 nm with a slit width of 5 nm. The voltage was set to 700 V. Aliquots were analyzed ex situ in quartz cuvette at times 48 and 144 h. The fluorescence intensity is normalized to higher fluorescence intensity obtained (in all cases the solution of lysozyme in the presence of Al­(III)).

#### 2.13.3 Fibrils Morphology Study by TEM

CEWL fibrillation samples were diluted 10-fold. A 5 μL drop of diluted solution was applied to carbon-coated copper grids for 30 min. The dried grids were stained with ammonium molybdate (1% w/v H_2_O).

## 3 Results and Discussion

### 3.1 Synthesis and Characterization of DSDA DFO-C12 and DFO@MSN

The initial objective of this work was to design and synthesize a precursor for MSN synthesis that incorporates a hydrocarbon chain into the DFO structure, thereby imparting amphiphilic properties in aqueous solutions (Figure S1). The formation of the amide derivative of DFO was confirmed by ^1^H, ^13^C NMR spectroscopy, FTIR analysis, MS, and elemental analysis. The ^1^H NMR spectrum of DFO-C12 (Figure S2) showed a signal at 5.39–5.28 ppm corresponding to the olefinic protons from the successfully incorporated aliphatic chain. Moreover, the signal at 2.99 ppm, associated with the protons nearest to the created amide bond, appears shifted due to the deshielding effect of the carbonyl group in the amide. The presence of a new amide group is evidenced by a proton signal at 7.77 ppm, which integrates for three protons. The ^13^C NMR spectrum (Figure S3) shows signals at 176 ppm, which confirms the formation of the amide through nucleophilic attack of DFO on the carbonyl group of the anhydride. Additionally, signals ranging from 133 to 127 ppm indicate the presence of double-bonded carbons belonging to the new carbon chain added to DFO. The FTIR spectrum (Figure S4) of DFO-C12 shows bands in the range of 2944–2847 cm^–1^, assigned to the stretching vibrations of the −CH_2_ and −CH_3_ groups of the hydrocarbon chain. It also exhibits a new band at 1711 cm^–1^ that corresponds to the amide moiety (Figure S5). The MS spectrum showed a molecular peak at 827.6 uma, confirming the formation of the desired product (Figure S5). To fully characterize the obtained molecule, we performed elemental analysis was performed. No differences were observed compared to the theoretically calculated amounts of C, H, N (Table S1). Surface tension measurements as a function of the DFO-C12 concentration were performed to determine the critical micellar concentration (CMC). The results showed a CMC of 0.6 mM for DFO-C12, indicating its suitability as a template for MSN synthesis (Figure S6).

After successfully obtaining DFO-C12 DSDA, the synthesis of MSNs was carried out based on previously established protocols,^39^ with modifications tailored to the properties of this DSDA, DFO-C12.

Above its critical micelle concentration, amphiphilic DFO-C12 (DSDA) self-assembles into anionic micelles that template silica condensation. Electrostatic complexation with the cationic costructure-directing agent (CSDA; protonated aminosilane APTES) drives the classic S^–^N^+^I^–^ pathway, wherein silicate/TEOS condenses around DSDA–CSDA ion pairs to nucleate and grow the mesostructure. This coassembly embeds DFO moieties within the silica framework (Figure 2A). This strategy allows MSNs to be synthesized and loaded with drugs simultaneously, eliminating the need for conventional postsynthesis loading steps.^39^ Nonetheless, we carried out a parameter study to fine-tune the nanoparticle size, which, as previously mentioned, is critical for nanoparticles intended for central nervous system applications.

**Figure 2:**
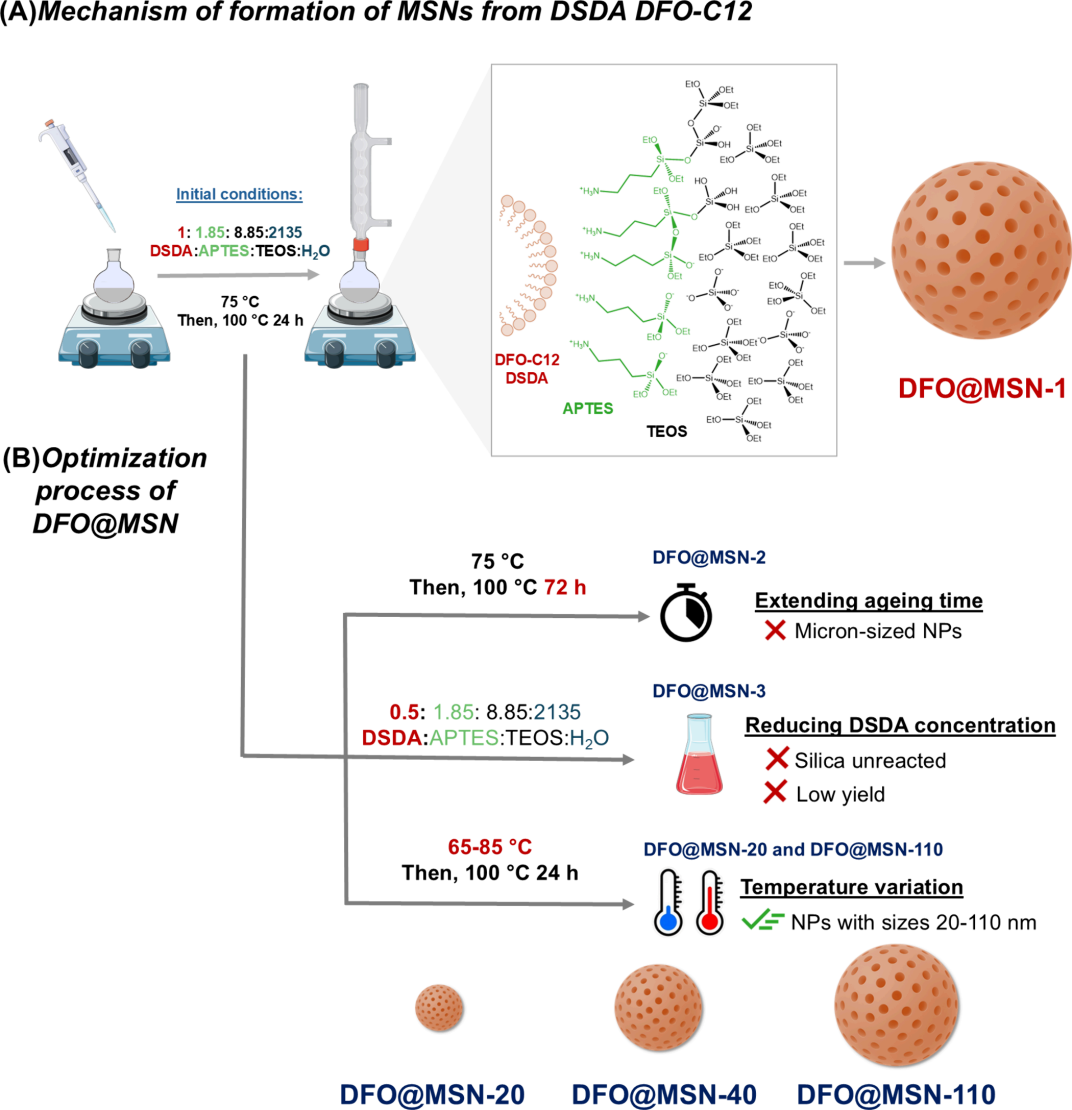
Schematic representation of (A) mechanism formation of DFO@MSN and (B) optimization process. Original scheme adapting images from Servier Medical Art https://smart.servier.com/ licensed under CC BY 4.0 (https://creativecommons.org/licenses/by/4.0/).

To optimize the synthesis of DFO@MSN and generate nanoparticles within desired specifications for drug delivery in the CNS, three key parameters were systematically investigated: aging time, DSDA concentration, and synthesis temperature (Figure 2B). Under the initial conditions, the resulting nanoparticles exhibited an average diameter of approximately 50 nm (DFO@MSN-1, Figure S7). Nitrogen adsorption–desorption (BET) analysis confirmed the mesoporous nature of the material, while thermogravimetric analysis (TGA) verified the successful incorporation of the drug (Table S2).

The first parameter evaluated was the aging time. Extending the duration from 24 to 72 h resulted in the formation of micron-sized particles (DFO@MSN-2), which are too large for drug delivery applications^31^ due to their increased size (Figure S7). Subsequently, the effect of the DSDA concentration was assessed. The standard synthesis protocol employed a surfactant concentration (26 mM) that was significantly higher than the CMC of DFO-C12. Therefore, a reduction of up to half of the concentration was explored (DFO@MSN-3). While this modification yielded nanoparticles within the desired size range, it also resulted in the formation of amorphous aggregates, likely composed of unreacted silica (Figure S8). BET and TGA analyses indicated a slight increase in both the porosity and organic content. However, the overall synthesis yield decreased markedly to 13% (Table S3). Due to the low yield and the presence of amorphous silica, this approach was deemed unsuitable and subsequently discarded.

Finally, the effect of the temperature on the synthesis process was evaluated. Figure 3 shows TEM images of DFO@MSNs synthesized at different temperatures. Notably, decreasing the temperature from 75 to 65 °C resulted in a fall in nanoparticle diameter to as low as 20 nm at 65 °C (DFO@MSN-20). Conversely, increasing the temperature resulted in larger and coalesced nanoparticles, ranging in size from 40 ± 6 nm for synthesis performed at 75 °C (we refer to this sample DFO@MSN-40 from here onward) to 110 ± 20 nm for synthesis at 85 °C (DFO@MSN-110), probably due to an increase in the nucleation kinetic constant. DLS measurements were conducted to confirm these results. The size distribution obtained follows the same trend as that observed by TEM (Figure 3A–C). These results are in consistent with previous studies on MCM-41-type materials, in which lowering the temperature resulted in smaller materials.^55,56^ As previously mentioned, the size of the nanoparticles is crucial when targeting the CNS. While there is no consensus on the ideal size, it is generally accepted that nanoparticles must be smaller than 100 nm to cross the BBB.^31^ Moreover, to allow for prolonged circulation in the bloodstream, the optimal size range is between 10 and 200 nm. Nanoparticles larger than 200 nm are rapidly cleared by the immune system, while those smaller than 10 nm are quickly excreted by the kidneys.^57^ Temperature variation enables us to optimize the nanoparticle size, generating uniform, monodisperse spheres ranging from 20 to 110 nm. To assess the distribution of the drug within MSNs synthesized from DSDA DFO-C12, EDS mapping was conducted (Figure S9). As shown in Figure 3D, the elemental maps of Si, O, C, and N confirm the homogeneous incorporation of organic moieties throughout the mesoporous silica framework.

**Figure 3:**
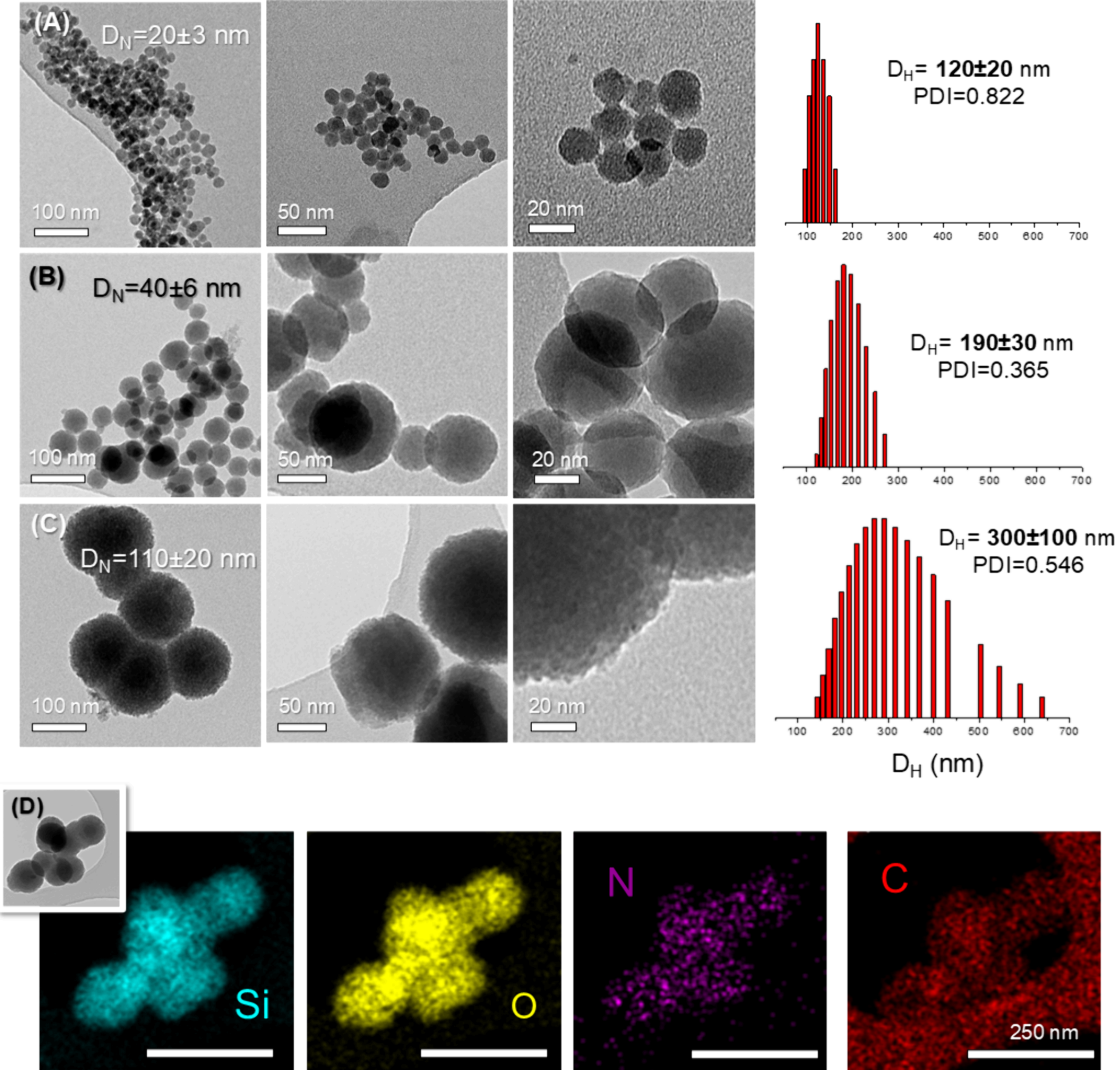
TEM images and DLS measurements performed at (A) 65 °C (DFO@MSN-20), (B) 75 °C (DFO@MSN-40), and (C) 85 °C (DFO@MSN-110). (D) EDS mapping analysis of a DFO@MSN-110. Scale bar = 250 nm.

The textural properties were calculated from the N_2_ adsorption isotherms of the calcined samples (Table 2). According to the IUPAC classification, all isotherms show the type IV curve (Figure 4), with a first capillary step at p/p_0_ = 0.4, which is typical of mesoporous materials normally used as DDS, such as MCM-41. BET analysis revealed similar specific surface areas for the materials synthesized at different temperatures. Pore size distribution curves show very narrow distributions centered at 3 nm, indicating that the entire porosity of the material originates from the use of DSDA-DFO-C12 as a template in MSN synthesis. Additionally, DFO@MSN-20 shows a hysteresis loop at high relative pressure (p/p_0_ = 0.9), which is consistent with the peaks shown at 5 and 10 nm in the PSD. This could be due to interparticle adsorption between the nanoparticles necks (Figure 4A), which are visible in some of the TEM images (Figure 3A). These results are consistent with the polydispersity index (PDI) obtained by dynamic light scattering (DLS) measurements. The PDI value for DFO@MSN-20 is much higher than that for DFO@MSN-40 (Figure 3A–C). Additionally, DFO@MSN-40 exhibits the lowest degree of aggregation, which can also be seen in TEM images, where monodisperse spheres with well-defined boundaries are observed (Figure 3C).

**Table 2:** Textural properties of DFO@MSN^a^

Nanomaterial	BET surface (m^2^ ·g^–1^)	Vp^a^ (cm^3^ ·g^–1^)	Dp^b^ (nm)	D_N_ ^c^ ± SD (nm)	LC% ± SD	EE% ± SD	Z-potential (mV)
DFO@MSN-20	241.1	0.4	3.1	20 ± 3	12 ± 3	11.8 ± 0.1	–22 ± 4
DFO@MSN-40	309.8	0.24	3.0	40 ± 6	10 ± 1	10.3 ± 0.4	–26 ± 8
DFO@MSN-110	273	0.23	3.1	110 ± 20	10 ± 4	9.4 ± 0.9	–15 ± 2

a DFO loaded is expressed as LC% and EE%.

b Vp: Pore volume.

c Dp: Pore size.

d D_N_: Nanoparticle size measured by TEM.

**Figure 4:**
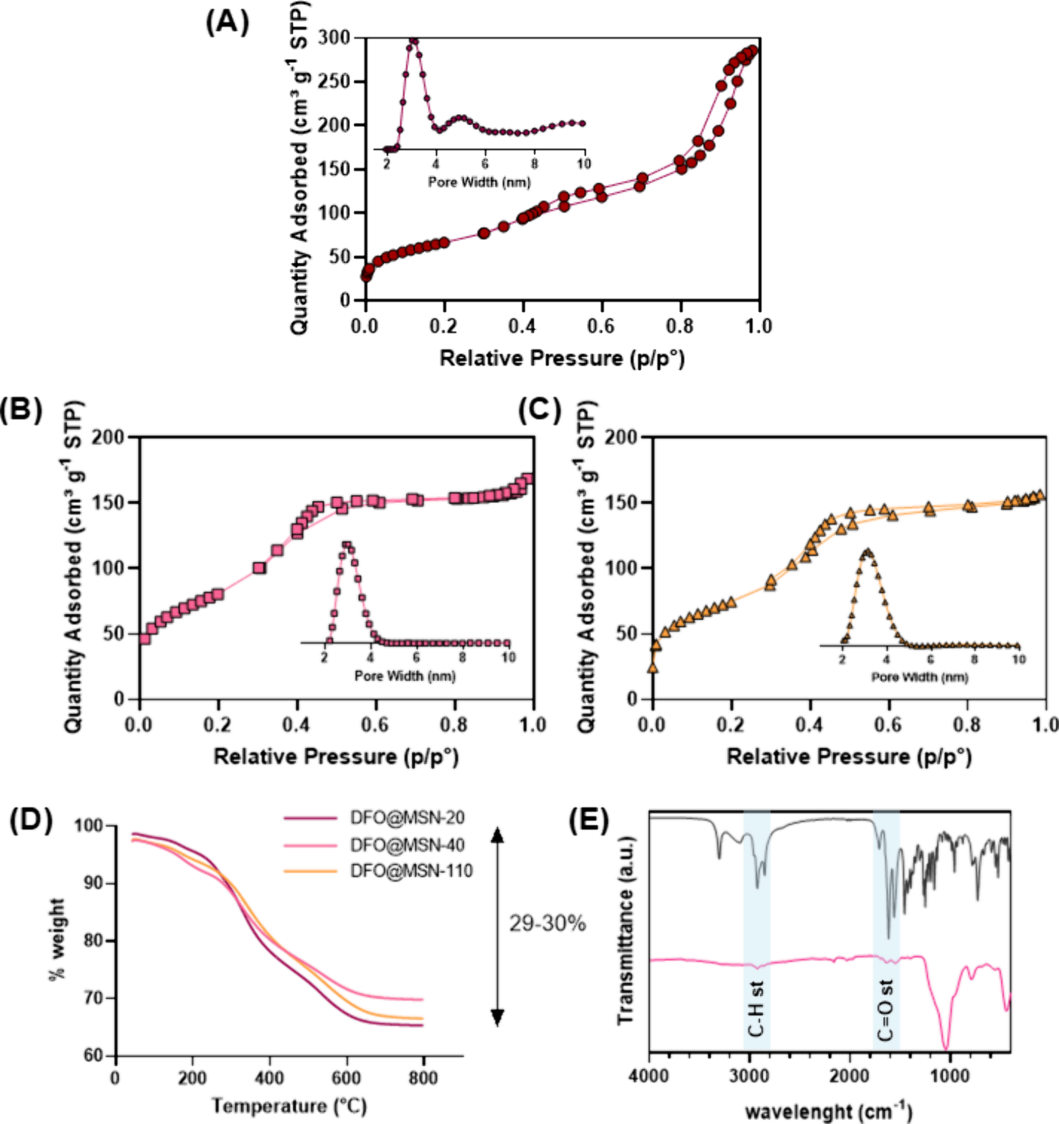
N_2_ adsorption isotherms and PSD graphs for (A) DFO@MSN-20, (B) DFO@MSN-40, and (C) DFO@MSN-110. (D) TGA of DFO@MSNs samples. (E) FTIR spectra of DSDA DFO-C12 (in gray) and DFO@MSN-40 (in pink).

The successful incorporation of the drug into the silica material was corroborated by FTIR spectroscopy and TGA (Figure 4D, E). The weight loss observed (Figure 4D) corresponds to CSDA and DSDA (Figure S10). Table 2 shows that the LC% and EE% determined by TGA Analysis of all samples indicates that the temperature of the synthesis does not affect the amount of DFO incorporated into MSNs (Figure 4D). The FTIR spectrum (Figure 4E) displays a band from 2950 to 2850 cm^–1^ due to hydrocarbons attached to DFO. Interestingly, a band that corresponds to the amide of DFO-C12 DSDA at 1711 cm^–1^ can be identified. Additionally, the spectrum shows the typical vibrations of −Si–O–Si– bonds at 1050 and 770 cm^–1^. To complete the characterization, XRD measurements of calcinated samples were carried out (Figure S11). The diffractograms showed no significant well-resolved XRD peaks, but an incipient reflection at 2θ = 2° confirms the mesoscopic ordering.

While the DSDA-based synthesis has proven reproducible in terms of nanoparticle size and drug loading capacity at laboratory scale, transitioning to larger production volumes will require optimization of mixing procedures and rigorous temperature control to ensure reproducibility. Overall, the synthetic refinements enabled the one-pot generation of monodisperse, size-tunable DFO@MSNs, establishing a clear temperature-size relationship that will guide the rational selection of nanoparticle formulations for optimal in vitro and BBB-permeable therapeutic performance.

### 3.2 *In Vitro* and Biological Characterization of DFO@MSN

As previously discussed, one of the main limitations of conventional drug encapsulation strategies lies in the requirement for favorable interactions between the silica matrix and the drug molecule to prevent premature release. This constraint becomes particularly critical for poorly soluble drugs, which are typically adsorbed onto the silica surface from highly concentrated solutions, often resulting in surface overloading and insufficient retention within the porous structure.^37^ To investigate the drug delivery behavior of this DFO formulation based on DSDA-MSN synthesis, release experiments were conducted using nanoparticles synthesized at various temperatures. In addition, a conventional DFO adsorption process was performed on a reference mesoporous material to compare it to the new encapsulation strategy. To determine the optimal adsorption conditions, two DFO concentrations, 7 and 15 mg·mL^–1^ (experiments A and B, respectively), were tested. The DFO loading capacity was also evaluated in two reference mesoporous silica nanoparticles with opposite surface charges: one functionalized with amino groups (MCM-41-NH_2_) and another with carboxylate groups (MCM-41-COOH). The quantity of DFO that was adsorbed onto the silica surface was measured and subsequently analyzed using TGA presented in Table S4. Among all the tested materials, the reference sample showing the highest drug loading (LC = 6.9%; EE = 4.6%) was DFO@MCM-41-COOH, which was selected for comparison. This result suggests a stronger interaction between DFO and acidic surface groups (carboxylates) than that with basic amino groups, which likely contributes to the increased amount of drug adsorbed. The DSDA-based synthesis strategy achieved significantly higher drug loading (LC = 10.1%; EE = 10.3%) representing a notable improvement over the traditional adsorption method. This substantial increase highlights the potential of the DSDA approach for the design of more efficient drug delivery systems.

The release profiles of the materials synthesized by the two different strategies, conventional surface adsorption and DSDA-based incorporation of DFO within the silica matrix, were markedly different (Figure 5). The reference material, in which the drug was adsorbed onto the surface, exhibited a typical burst release behavior, with 75% of the DFO released in less than 24 h. In contrast, DFO@MSN-40, where the drug was incorporated into the pores during synthesis, showed a much slower and sustained release profile: less than 20% of the drug was released after 24 h, and approximately 60% was released over the entire duration of the experiment (390 h). The data underscore the limitations of the traditional encapsulation approach, as most of the drug appears to be adsorbed on the external surface of the silica particles, which likely contributes to its premature release.^41^ Conversely, the DSDA method is characterized by its ability to fill the pores, thereby ensuring sustained and prolonged release.

**Figure 5:**
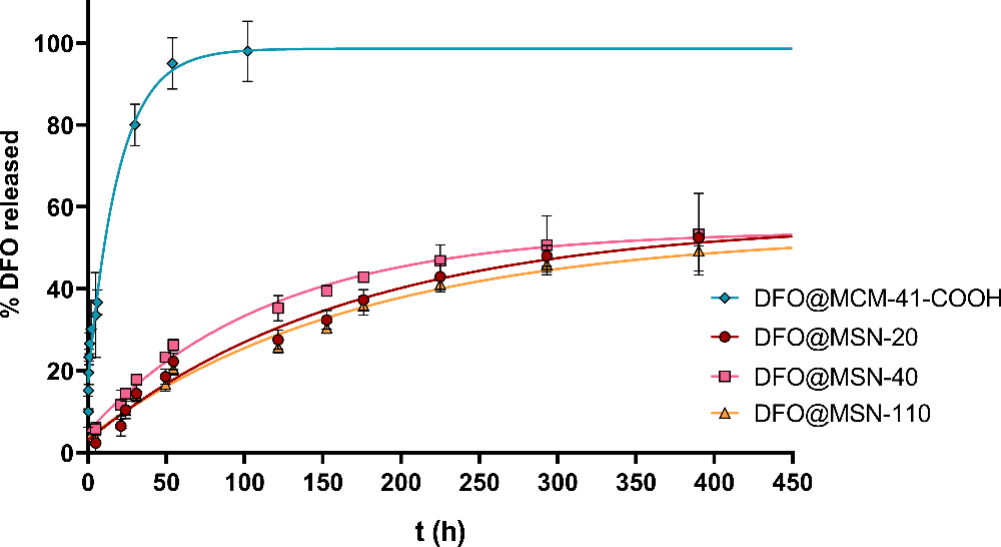
Release study of DFO at pH = 7.4 from reference material (in blue) and DFO@MSN-20 (red), DFO@MSN-40 (in pink), and DFO@MSN-110.

Release profiles were fitted to the Higuchi and Korsmeyer–Peppas models. The corresponding k, n, and R^2^ values are reported in Table S5. DFO@MSN formulations exhibit lower Higuchi and Korsmeyer–Peppas rate constants than DFO@MCM-41-COOH, confirming their slower, more sustained release. Moreover, the n values for DFO@MSN-20, DFO@MSN-40, and DFO@MSN-110 (0.52–0.69) indicate anomalous, non-Fickian transport governed by a combination of diffusion through the mesoporous silica and specific drug–silica interactions/constraints effects from DFO embedded via the DSDA strategy, whereas the n value for DFO@MCM-41-COOH (0.32) is consistent with Fickian diffusion and the pronounced burst release observed for the surface-adsorbed DFO.

After the release profile was studied, the safety of the nanoparticles was evaluated using the BV-2 and SH-SY5Y cell lines. BV-2 is a microglial cell line that is particularly relevant for evaluating compounds intended for the treatment of neurodegenerative diseases, as these disorders are well-known to involve chronic inflammation. In BV-2, a 40% decrease in cell viability was observed only for DFO@MSN-20 at 100 μM, but no significant differences were found between the formulations at this concentration. Moreover, such high concentrations are unlikely to occur in the brain due to the sustained release profile of the DFO@MSNs and the brain clearance mechanisms.^58^ The slight decrease in viability may be attributed to membrane disruption after 72 h of exposure.^59^ Nevertheless, DFO-C12 and DFO@MSNs did not induce cellular damage across the tested concentrations (25–100 μM) (Figure 6), showing no statistical differences compared with free DFO. MCM-41 nanoparticles were used as a control (Figure S12A), and the results confirmed the biocompatibility of the synthesized materials, consistent with previous studies.^47,60^ To broaden the safety assessment beyond microglia, we evaluated cytocompatibility in the human neuroblastoma line SH-SY5Y, a neuronal-like model widely used in neuroprotection studies. SH-SY5Y assays confirmed the overall biocompatibility of the MSN-based materials up to 100 μM (Figure S13). In agreement with BV-2 results, viability at 100 μM was ∼80% for DFO-C12 and ∼100% for free DFO. Among the MSN formulations, DFO@MSN-20 and DFO@MSN-40 showed the largest viability reductions (both ∼70% at 100 μM), consistent with the BV-2 trend (DFO@MSN-20 ∼60% at 100 μM). Cell viability as a function of nanoparticle concentration and MCM-41 control is represented in Figure S12B.

**Figure 6:**
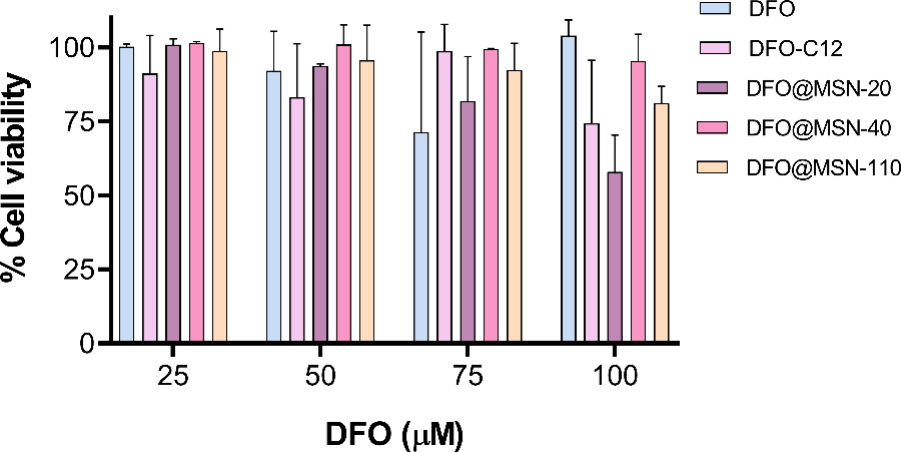
Cell viability assays conducted in BV-2 at 72 h.

To assess the therapeutic potential of DFO-C12 and DFO@MSNs, Fe­(III) chelation assays were performed. The formation of the Fe­(III) complex with DFO-C12 was first investigated in homogeneous phase using the method of continuous variations (Job’s method, Figure S14). The stoichiometry of the Fe­(III)-DFO-C12 complex was determined to be 1:1, which is consistent with that of unmodified DFO and with previous reports.^61^ This result indicates that the chelating ability of DFO is preserved after modification with the hydrophobic C12 chain, which is required for DSDA formation. The strong chelating ability of DFO is due to its three bidentate hydroxamate groups, which form highly stable complexes with metal ions.^20^

The chelation capacity of the nanoparticles was also evaluated. To this end, DFO@MSN-40 was incubated in a concentrated Fe­(III) solution. Energy-dispersive X-ray spectroscopy (EDS) mapping analysis (Figure 7A) revealed a homogeneous distribution of Fe across the silica surface, indicating successful complexation. Following the assay, the nanoparticles were dissolved, and their iron content was quantified by ICP-OES. As shown in Figure 7B, DFO@MSN-40 displayed a higher Fe content compared to the reference material, suggesting that the DFO-C12 units embedded within the mesopores effectively capture iron from the surrounding environment.

**Figure 7:**
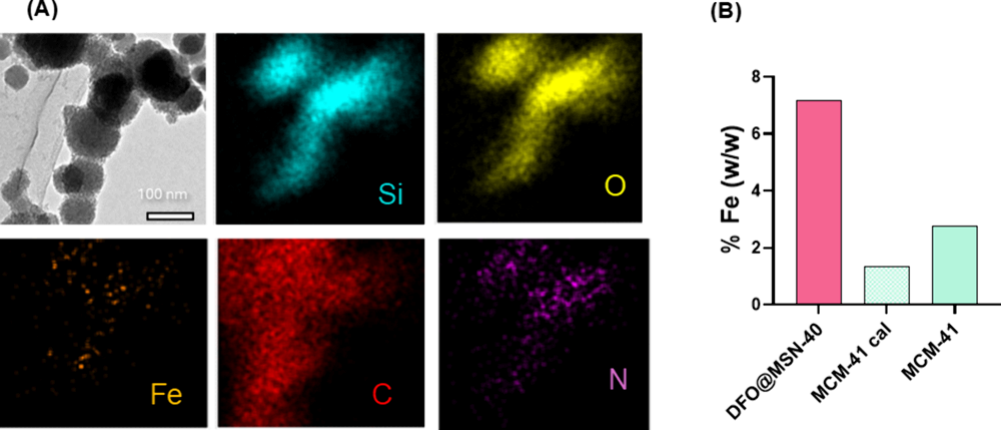
(A) EDS mapping of DFO@MSN-40 after chelation assay. (B) Fe content determined by ICP-OES after chelation assay using DFO@MSN-40 and reference material calcined (MCM-41 cal) and noncalcined (MCM-41) as controls.

The effect of DFO@MSN on metal-induced protein aggregation was investigated by fluorescence and TEM microscopy. Lysozyme was chosen as a model for β-Amyloid-like aggregation because both proteins share the same folding mechanism.^53^ To detect fibril formation, ThT was used as an indicator since a increment in fluorescence intensity is produced in the presence of β-sheet-rich structures and it has been widely used as a detector of amyloid-like fibrils.^52^ Amyloid fibrillation was induced under thermal and acidic conditions according to previously published protocols.^52,53^ The effect of metals on misfolding is first examined by measuring ThT fluorescence in samples incubated with Fe­(III) and Al­(III). As shown in Figure 8A, only Al­(III) increases the fluorescence, indicating its effect on promoting fibril aggregation. Previous studies confirm that aluminum promotes lysozyme fibrillation under heat-induced conditions, while Fe­(III) has not been identified as a contributor. However, substantial evidence implicates iron in protein misfolding and ND progression.^11,19^ The absence of increment of fluorescence simply suggests that misfolding is a complex process involving several intermediates that may not form β-sheet structures in the presence of iron.^13^ Based on these findings, the effect of DSDA DFO-C12 and DFO@MSN-40 on Al­(III)-induced amyloid fibrillation was evaluated. Before evaluating the treatments, we monitored lysozyme aggregation in the presence of Al­(III) under the assay conditions. The ThT time course exhibited a sigmoidal profile with a lag phase, a growth phase, and an equilibrium phase (Figure S15). Guided by this kinetic profile, treatments were introduced at 48 h (late lag/early growth, enriched in prefibrillar intermediates) and assessed again at 144 h (equilibrium phase, enriched in mature fibrils). This design allows us to distinguish effects on nucleation/early elongation versus late-stage fibril burden. After 48 h (Figure 8B), DSDA DFO-C12 appears to inhibit amyloid fibril formation, as fluorescence intensity correlates with concentration of fibrils *in vitro*. Similarly, DFO@MSN-40 reduces fluorescence compared to the sample incubated only with Al­(III), indicating a similar inhibitory effect. These results may be related to the capture of the Al­(III) ions by the chelating agent. This effect cannot be attributed solely to DFO, as MCM-41 (reference material) also reduces fibril formation, implying that the interaction between the silica surface and lysozyme may play a role in preventing β-sheet structures. This phenomenon has been previously reported and attributed to lysozyme adsorption onto the silica surface.^62^ Similarly, polymeric nanoparticles exhibit comparable inhibitory effects on protein aggregation, specifically by delaying the nucleation phase without affecting elongation.^63^ Finally, the time-dependent effect of the treatments was evaluated. It is noteworthy that the inhibitory activity persisted over time, as evidenced by the sustained reduction in fluorescence intensity observed at 144 h in samples treated with DSDA and MSNs compared to the Al­(III)-control (Figure 8C). In addition, the DFO@MSN-40 sample exhibited significantly diminished fluorescence in comparison to the MCM-41 reference, indicating a probable therapeutic effect likely associated with the sustained release of DFO.

**Figure 8:**
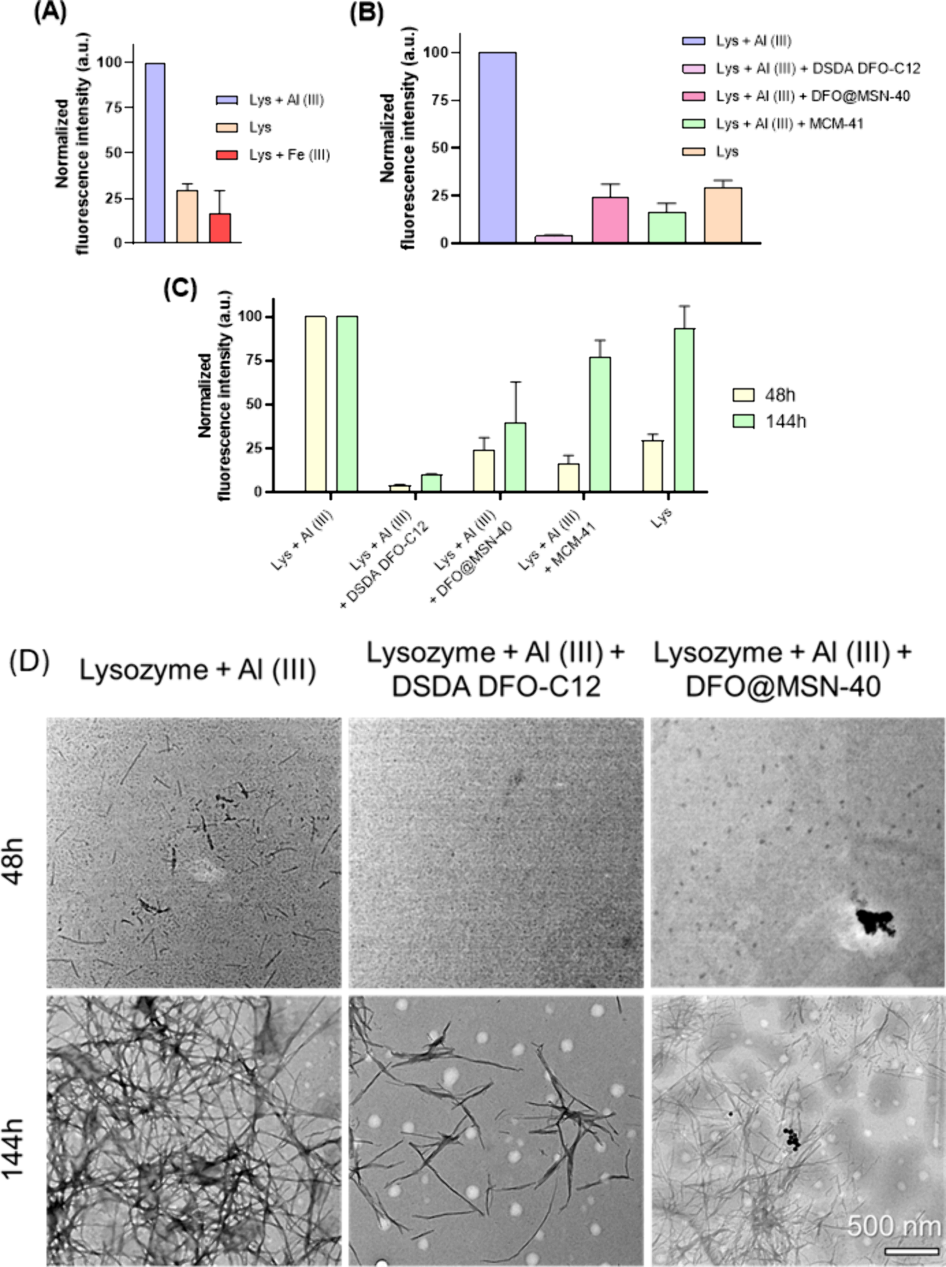
(A) ThT fluorescence intensity of incubated samples at pH = 1.6 and 65 °C in the presence of Fe­(III) and Al­(III) at 48 h. (B) ThT fluorescence intensity of incubated samples at pH = 1.6 and 65 °C in the presence of Al­(III) and treatments at 48 h. (C) ThT fluorescence intensity over the time. (D) TEM images of lysozyme fibrils incubated in the presence of Al­(III), Al­(III) and DSDA DFO-C12 and Al­(III) and DFO@MSN-40 at 48 and 144 h.

The negative staining TEM was employed to investigate the morphology of the protein aggregates in the presence of metals and chelators. At 48 h (Figure 8D), the Al­(III) sample exhibited the presence of small fibrils; however, these were not discernible in samples that had been incubated with chelators. By 144 h, the concentration of fibrils was found to be significantly lower in the presence of DFO-C12 and DFO@MSN-40 compared to Al­(III). Supplementary TEM images illustrating the folding process are provided in Figure S16. Additionally, the fibrils that underwent incubation with DSDA DFO-C12 and DFO@MSN-40 exhibited a greater degree of fragmentation compared with the control experiment. The elongation rate is known to increase in response to elevated levels of ionic strength in the solution.^64^ These findings are consistent with the ThT assay results. Therefore, the presence of chelators capturing metals could play a role in slowing the formation of these aggregates. The nanoformulation, in addition to functioning as a chelator, could have an indirect therapeutic effect by inhibiting the formation of these widely prevalent aggregates in various neurodegenerative diseases.

Finally, the capacity to penetrate the BBB for DSDA DFO-C12 and DFO@MSNs was evaluated. As previously stated, a primary challenge associated with the utilization of DFO as a therapeutic agent for ND is its low bioavailability. Amide derivatives have been reported as a method to enhance the hydrophobicity of DFO,^65^ promoting the cellular internalization of DFO^66^ and demonstrating neuroprotective effects in an animal PD model.^67^ Considering the previous findings, it can be hypothesized that the synthesis of DFO-C12 may lead to an increase in the bioavailability of DFO. For the DSDA DFO-C12 study, the data obtained from log P (both calculated and experimental) was analyzed, as well as the permeability coefficient determined using an *in vitro* cell-based BBB model. The log P value of the synthesized compound was determined using RP-HPLC. As shown in Table 3, DFO-C12 DSDA exhibits a log P value of 1.37, indicating a higher lipophilic character compared to the parent molecule (log P = −0.02). In contrast, DFO exhibits a higher solubility in the aqueous phase, which may be an additional challenge for its internalization across the cell membrane.

In addition, to support this hypothesis, BBB permeability assays were performed by using the Caco-2 cell line. This line is frequently utilized *in vitro* BBB models.^68^ Following a 21 day culture period, Caco-2 cells undergo the development of tight junctions and the expression of membrane transporters that are analogous to those observed in the BBB. The phenomenon of drug efflux across the BBB is mediated by multiple transporters, primarily belonging to the ATP-binding cassette (ABC) family. Among these, P-glycoprotein (P-gp) is the most extensively studied and widely recognized efflux transporter. Drug interaction with the BBB is thus influenced not only by passive membrane permeability but also by the activity of efflux pumps, such as P-gp, breast cancer resistance protein (BCRP), and multidrug resistance-associated proteins (MRPs), which collectively contribute to limiting drug penetration into the central nervous system. Since P-gp is predominantly expressed on the apical side of the cellular monolayer, compounds with high affinity for this efflux transporter tend to accumulate in the apical compartment. This limits their ability to cross the BBB.^69,70^ As a result, the apical-to-basolateral permeability (Papp AB), which reflects transport from the blood side toward the brain, is often reduced and is considered indicative of ″active transport restriction″ due to efflux pump activity. In contrast, the basolateral-to-apical transport (Papp BA), which reflects movement from brain to blood, is largely driven by passive diffusion and is less affected by efflux mechanisms. The BA/AB permeability ratio is a useful parameter to assess the overall impact of efflux transporters. BA/AB ratio close to 1 suggests symmetrical transport, likely governed by passive diffusion; BA/AB ratio significantly greater than 1 indicates preferential efflux from the basolateral to the apical side, suggesting that active efflux transporters, such as P-gp, are limiting the apical-to-basolateral transport (i.e., entry into the brain or tissue); BA/AB ratio <1 may suggest active uptake or other nonefflux phenomena. Thus, the BA/AB ratio provides insight into whether a compound’s transport is balanced, efflux-dominated, or influenced by other mechanisms.

As shown in Table 3, DFO shows a BA/AB ratio of 2.13, indicating significant asymmetry in its transport across the monolayer. This suggests that active efflux mechanisms, such as P-gp, are likely to limit its apical-to-basolateral (blood-to-brain) permeability. The high efflux ratio is consistent with DFO being a likely substrate of efflux transporters, which could reduce its brain penetration. DFO-C12, on the other hand, displays a BA/AB ratio of 1.19, much closer to unity. This suggests a more balanced bidirectional transport, with **r**educed contribution from active efflux. The increased Papp AB value compared to DFO further supports the notion that C12 may have improved passive permeability and/or decreased transporter-mediated efflux. In summary, the C12 modification appears to attenuate efflux susceptibility, potentially enhancing the compound’s ability to cross the BBB.

**Table 3:** Log P Values and Permeability Coefficients^a^

Compound	Log P (calculated)	Log P (experimentally obtained)	Papp BA (nm·s^–1^)	Papp AB (nm·s^–1^)	BA/AB
DFO	–0.03	–0.02	1619	761	2.13
DFO-C12	1.71	1.37	1482	1270	1.19

a Log P values calculated using http://www.swissadme.ch. Permeability coefficients calculated in an *in vitro* cell-based BBB model.

Finally, the ability of DFO@MSNs to cross the BBB was evaluated using a Caco-2 cell monolayer model in order to assess whether nanoparticle size optimization could significantly enhance brain-targeted drug delivery. This evaluation is particularly relevant because, although the addition of a hydrocarbon chain improves the BBB permeability of DFO-C12, its apparent permeability remains insufficient to classify it as BBB-permeable.

To quantify the transport of DFO@MSNs across the monolayer, nanoparticles were fluorescently labeled with FITC and subjected to transport experiments following a previously established protocol,^51^ measuring their movement from the apical (top) to the basolateral (bottom) chamber. Since MSNs are significantly larger than the binding pocket of efflux transporters such as P-gp, their transport is not affected by active efflux mechanisms. Therefore, the observed permeability exclusively reflects the passive diffusion across the monolayer.

To distinguish this from classical small-molecule permeability, we define this parameter as Papp^NP^_AB (apparent permeability for nanoparticles, apical-to-basolateral). This notation emphasizes that the measured transport is independent of active transporter interactions and specifically refers to nanoparticle diffusion. A schematic of the experimental setup is shown in Figure 8A and B. A concentration of 50 μg·mL^–1^ was used in all experiments to prevent cytotoxicity and preserve the monolayer integrity (Figure S17).

As shown in Figure 9B, a clear size-dependent permeability was observed. The smallest nanoparticles, DFO@MSN-20, exhibited the highest apparent permeability coefficient (Papp^NP^_AB = 761 nm·s^–1^), followed by DFO@MSN-40 (Papp^NP^_AB = 489 nm·s^–1^) and DFO@MSN-110 (Papp^NP^_AB = 343 nm·s^–1^), confirming that nanoparticle transport across biological barriers is strongly influenced by particle size.^31–33^

**Figure 9:**
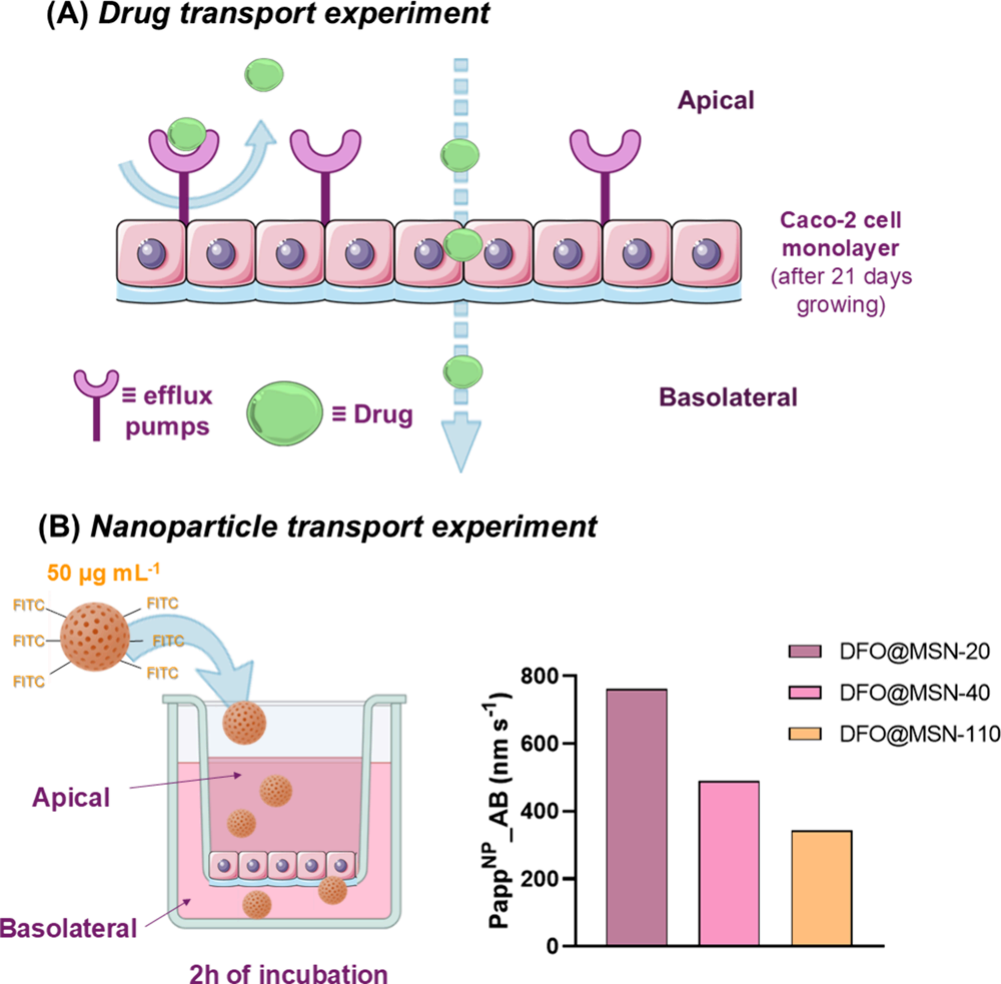
Schematic representation of (A) drug transport experiment from apical to basolateral, Papp AB (apparent permeability from basolateral to apical), and (B) nanoparticles transport experiment, Papp^NP^_AB (apparent permeability for nanoparticles, apical-to-basolateral) calculated for DFO@MSN-20, DFO@MSN-40, and DFO@MSN-110. Original scheme adapting images from Servier Medical Art https://smart.servier.com/ licensed under CC BY 4.0 (https://creativecommons.org/licenses/by/4.0/).

These findings underscore the particle size as a critical parameter in the rational design of brain-targeted drug delivery systems. Smaller particles demonstrated a superior ability to traverse the *in vitro* monolayer, suggesting enhanced potential for BBB penetration. However, it is important to note that unlike small-molecule drugs, where well-established thresholds for permeability classification exist, standardized benchmarks for nanoparticle permeability are still lacking. This limits direct comparison between different studies and hinders the establishment of predictive models. Therefore, continued investigation into structurally diverse nanocarriers and the refinement of *in vitro* BBB models are essential for improving the translational potential of nanomedicines. Most importantly, our data provide experimental support for the strategy of size reduction as a means to enhance BBB permeability, addressing one of the major challenges in CNS drug delivery.

In the context of current DFO nanoformulations reported in literature, dendritic polymers,^25^,^26^ targeted polymeric NPs,^27^,^28^ and 2D-nanosheets^29^ systems extend circulation and reduce toxicity but often require multistep syntheses, complexity and long-term stability. Only two studies have explored the use of MSNs for chelation therapy,^9^,^38^ however, both systems exhibited a burst drug release profile with a plateau within 24 h due to the weak drug-matrix interactions. Our DSDA-based DFO@MSNs unify (i) one-pot, surfactant-free drug incorporation, (ii) precise size-tuning from ∼20 to 110 nm (favoring BBB transport), and (iii) sustained release with <20% DFO liberated at 24 h, while maintaining mesoporosity and homogeneous drug distribution. Together with the observed size-dependent permeability across an epithelial barrier model, these attributes position DSDA-templated MSNs as a mechanistically rational alternative for brain-directed chelation therapy.

## Conclusions

The use of a DFO derivative as a drug-structure-directing agent (DSDA) has proven to be an effective strategy for developing nanometer-sized mesoporous silica nanoparticles (MSNs). By tuning the synthesis temperature, we achieved precise control over particle size, obtained MSNs ranging from 20 to 110 nm. DSDA-based synthesis enabled significantly higher drug loading compared with traditional adsorption methods. Moreover, the drug release profile of DFO@MSNs was sustained over time, with less than 20% of the encapsulated drug being released within the first 24 h. In contrast, DFO loaded via postsynthetic adsorption exhibited a burst release, with 75% of the drug released within 24 h. The safety of both the synthesized DFO-C12 derivative and the resulting DFO@MSNs was demonstrated *in vitro* using a BV-2 microglial cell line, showing no cytotoxic effects after 72 h, even at concentrations up to 100 μM.

The therapeutic potential of the DFO@MSNs platform was further supported by several *in vitro* studies. In particular, the iron-chelating ability of the DFO@MSNs confirmed the presence of surface-accessible DFO moieties. Additionally, the antiamyloidogenic activity of DFO-C12 and DFO@MSNs was evaluated using an aluminum-induced fibrillation assay. DFO-C12 significantly reduced amyloid fibril formation even after 144 h, as evidenced by fluorescence spectroscopy and TEM imaging. A similar inhibitory effect was observed for DFO@MSNs, which exhibited lower fluorescence intensity compared with the reference material at the same time point. Finally, BBB permeability studies using a Caco-2 cell monolayer demonstrated that decreasing MSN particle size substantially enhanced their ability to cross biological barriers, a critical feature for central nervous system (CNS)-targeted drug delivery.

Together, these findings demonstrate the successful design of a new generation of DFO-based nanocarriers capable of sustained drug release, efficient iron chelation, and BBB permeability in a size-dependent manner, thereby opening new avenues for DFO delivery in neurotherapeutic applications. Following the *in vitro* confirmation of iron-chelating and antiamyloidogenic activity, future work will evaluate DFO@MSNs in relevant *in vivo* models to assess their behavior under physiological conditions, including biodistribution, safety, and therapeutic efficacy.

## Supplementary Material



## Notes

NMR spectra; ESI mass spectrum; elemental analysis; textural properties; additional TEM images; EDS mapping analysis; TGA analysis; XRD patterns; kinetics parameters; additional cell viability assays (PDF)
