# Supplementary material for: Size-Controlled Mesoporous Silica Nanoparticles via Template Nanoarchitectonics from a Deferoxamine Derivative for Enhanced Blood–Brain Barrier Permeability and Neuroprotective Chelation Therapy
Source: ACS Appl Mater Interfaces. 2025 Dec 11;17(51):69090–106. doi: 10.1021/acsami.5c18528 (PMC12754760; doi:10.1021/acsami.5c18528)
Supplement: Supplementary file 1 [file am5c18528_si_001.pdf]

Supporting Information

Size-controlled mesoporous silica nanoparticles via template nanoarchitectonics from a deferoxamine derivative for enhanced blood-brain barrier permeability and neuroprotective chelation therapy

Authors: Mónica Onrubia-Márquez[a], Francisco Navas[a], Esther M. Sánchez-Carnerero[a] Antonio Martín[a], Anselma Liturri[c], Morena Miciaccia[c], Raúl Sanz[a,b], Antonio Scilimati[c], Rafael A. García-Muñoz[a,b], Maria Grazia Perrone[c], Victoria Morales[a*]

Affiliations:

a. Department of Chemical and Environmental Technology. ESCET, Universidad Rey Juan Carlos, 28933, Móstoles, Madrid, Spain.

b. Instituto de Investigación de Tecnologías para la Sostenibilidad, Universidad Rey Juan Carlos (URJC), 28933 Móstoles, Madrid, Spain.

c. Research Laboratory for Woman and Child Health, Department of Pharmacy-Pharmaceutical Sciences, University of Bari "Aldo Moro", 70125 Bari, Italy.

Characterization of DSDA DFO-C12

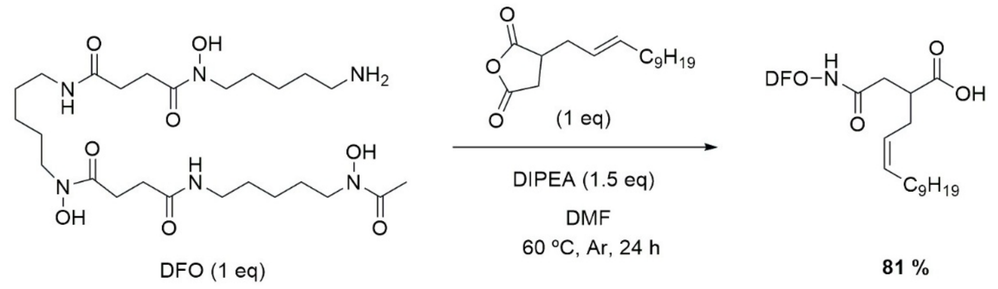

Figure S1. Synthesis of DFO-C12.

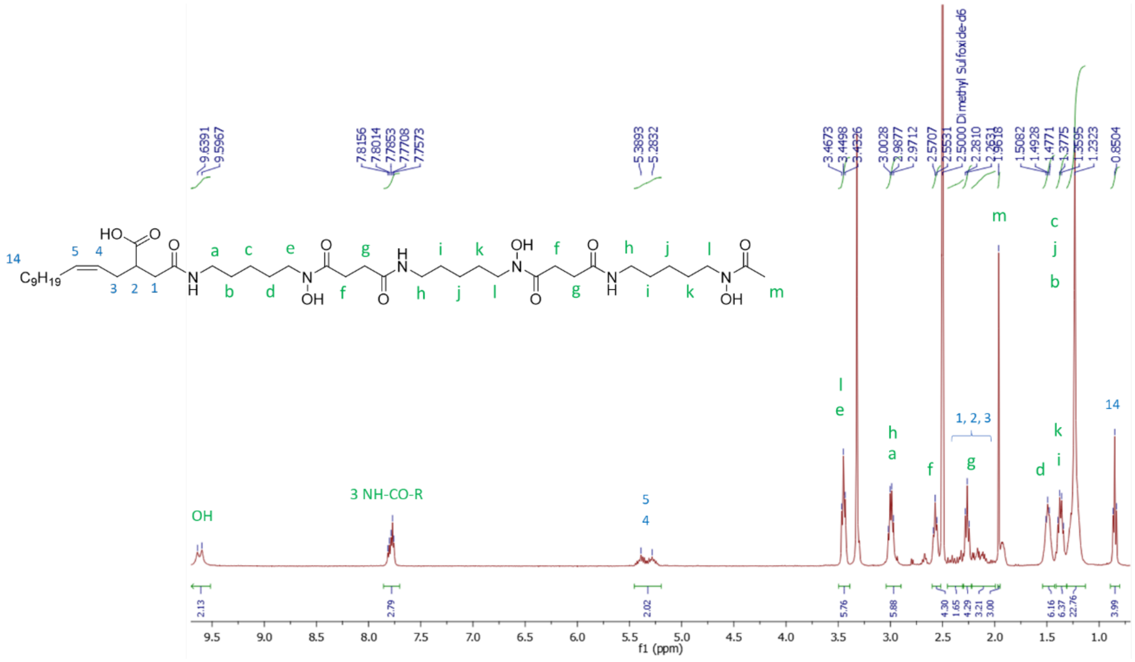

Figure S2. ^{1}H NMR of DSDA DFO-C12.

^{1}H-NMR (400 MHz, d$_6$-DMSO), *δ* (ppm): 9.61 (s.a., 2H, OH), 7.80 (t, J=5.7, 1H, NH), 7.77 (t, J= 5.4 Hz, 2H, NH), 5.38-5.28 (m, 2H, CH-4 and 5), 3.47-3.43 (m, 6H, CH$_2$-l and e), 3.02-2.97 (m, 6H, CH$_2$-h and a), 2.57 (t, J=7.2 Hz, 4H, CH$_2$-f), 2.26 (t, J=7.2 Hz, 4H, CH$_2$-g), 1.96 (s, 3H, CH$_3$-m), 1.51-1.48 (m, 6H, CH$_2$-d and k), 1.23 (m, 24H, CH$_2$-6-13,c, j and b), 0.85 (t, J=6.4 Hz, 3H, CH$_3$-14).

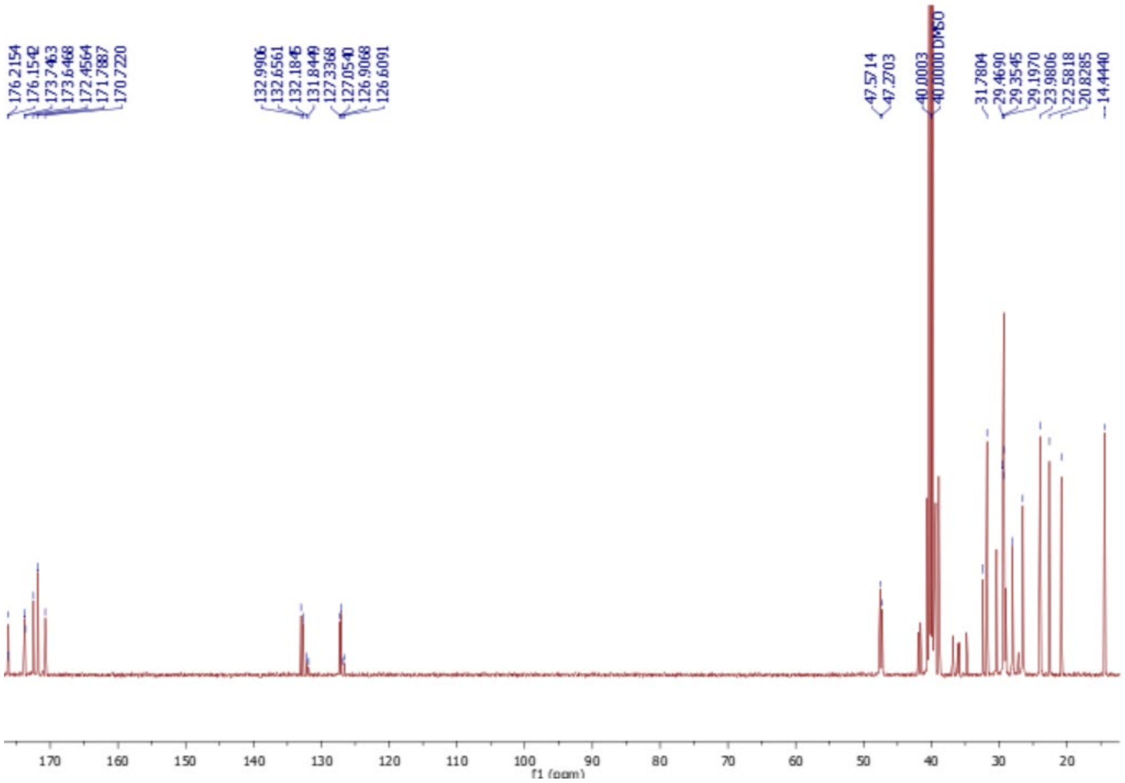

Figure S3. ^{13}C NMR of DSDA DFO-C12.

^{13}C-NMR (400 MHz, d_6-DMSO), δ (ppm):176.2 (C=O), 176.2 (C=O), 173.8 (C=O), 173.7 (C=O), 172.5 (C=O), 171.8 (C=O), 170.7 (C=O), 133.0 (CH), 132.7 (CH), 132.2 (CH), 131.8 (CH), 127.3 (CH), 127.1 (CH), 126.9 (CH), 126.6 (CH), 47.6 (CH_3), 47.3 (CH_3), 32.4 (CH_2), 31.8 (CH_2), 29.5 (CH_2), 29.4 (CH_2), 29.2 (CH_2), 28.1 (CH_2), 26.5 (CH_2), 24.0 (CH_2), 22.6 (CH_2), 20.8 (CH_2), 14.4 (CH_2).

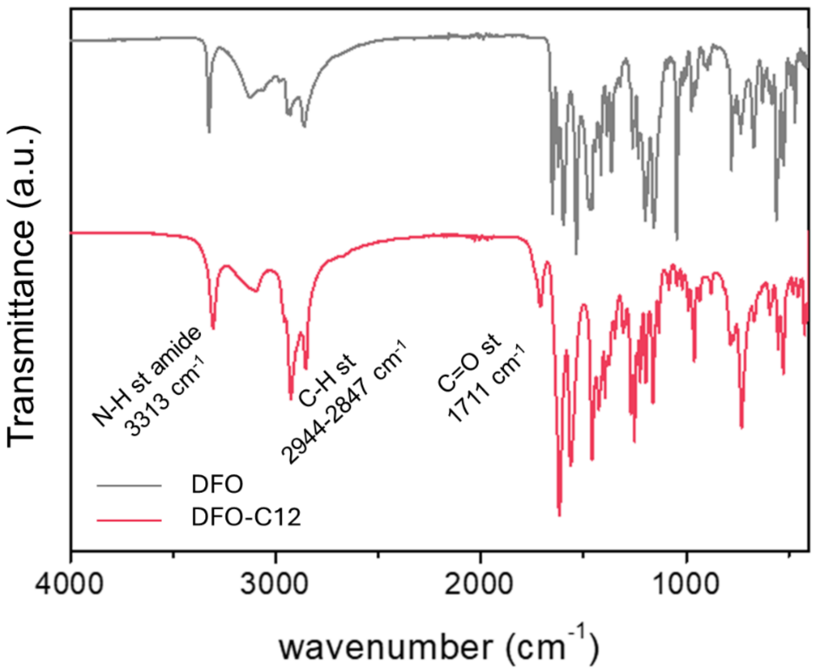

Figure S4. FTIR Spectrum of DSDA DFO-C12.

FTIR, ν: 3313 (N-H amide st), 3095 (O-H st), 2944 and 2847 (C-H st), 1711 (C=O st), 1624 (C=O st) cm^{-1}.

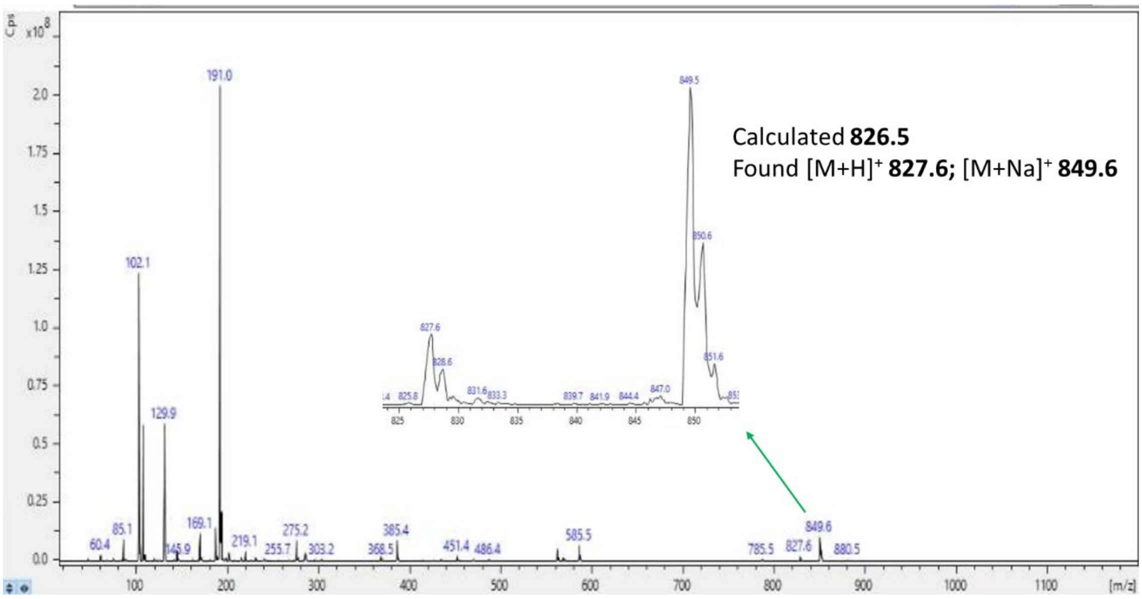

Figure S5. ESI Mass Spectrum of DFO DSDA. Calculated for $C_{41}H_{74}N_6O_{11}$ m/z=826.5. Found m/z= 827.6 $[M+H]^+$ and 849.6 $[M+Na]^+$.

Table S1. Elemental Analysis of DSDA DFO-C12.

	%N	%C	%H
**Calculated**	9.99	59.98	9.11
**Found**	10.78	58.52	9.12

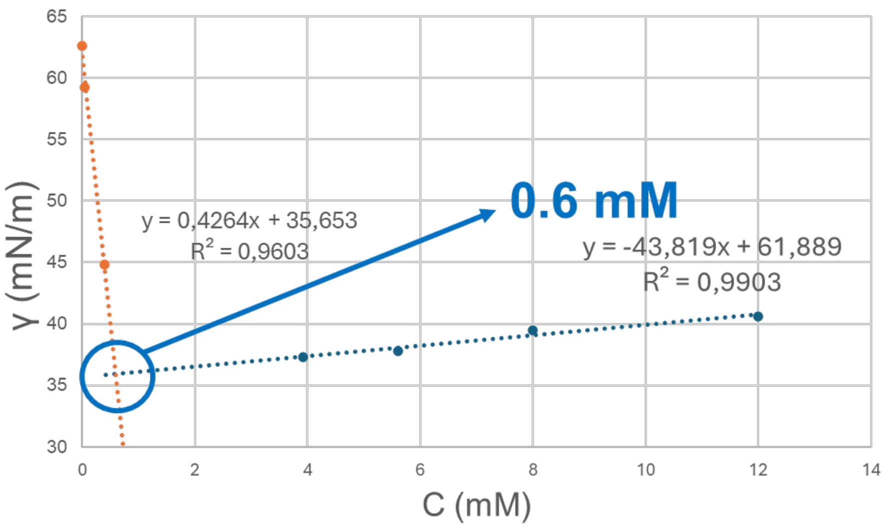

Figure S6. CMC of DSDA DFO-C12.

Characterization of DFO@MSN

Table S2. Textural properties of nanomaterials obtained from ageing time variation.

Nanomaterial	Ageing time (h)	BET surface ($m^2 \cdot g^{-1}$)	Vp ($cm^3 \cdot g^{-1}$)	Dp (nm)	wt % (TGA)	% Yield
DFO@MSN-1 (DFO@MSN-40)	24	309.8	0.24	3.0	29	19
DFO@MSN-2	72	457.5	0.47	2.6-2.8	45	16

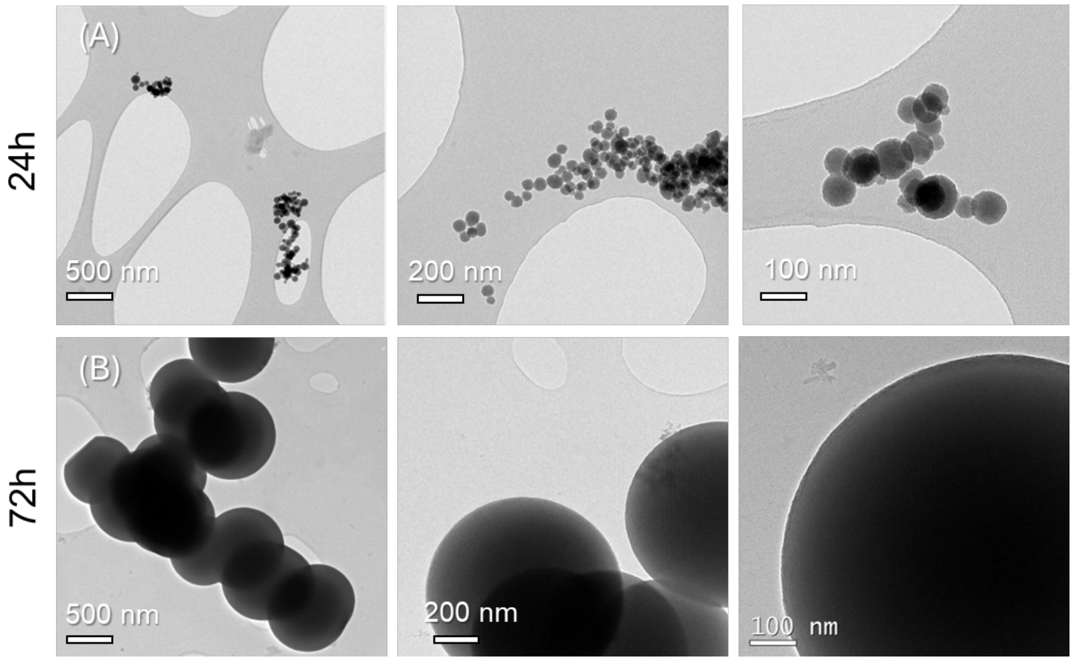

Figure S7. (A) TEM images of DFO@MSN-1 with aging time of 24h. (B) Synthesis of DFO@MSN-2 with aging time of 72h.

Table S3. Textural properties of nanomaterials obtained from DSDA concentration variation.

**Nanomaterial**	**[DSDA] (mM)**	**BET Surface ($m^2·g^{-1}$)**	**Vp ($cm^3·g^{-1}$)**	**Dp (nm)**	**wt % (TGA)**	**%Yield**
DFO@MSN-1 (DFO@MSN-40)	26	309.8	0.24	3.0	29	19
DFO@MSN-3	13	491.4	0.42	3.1	35	13

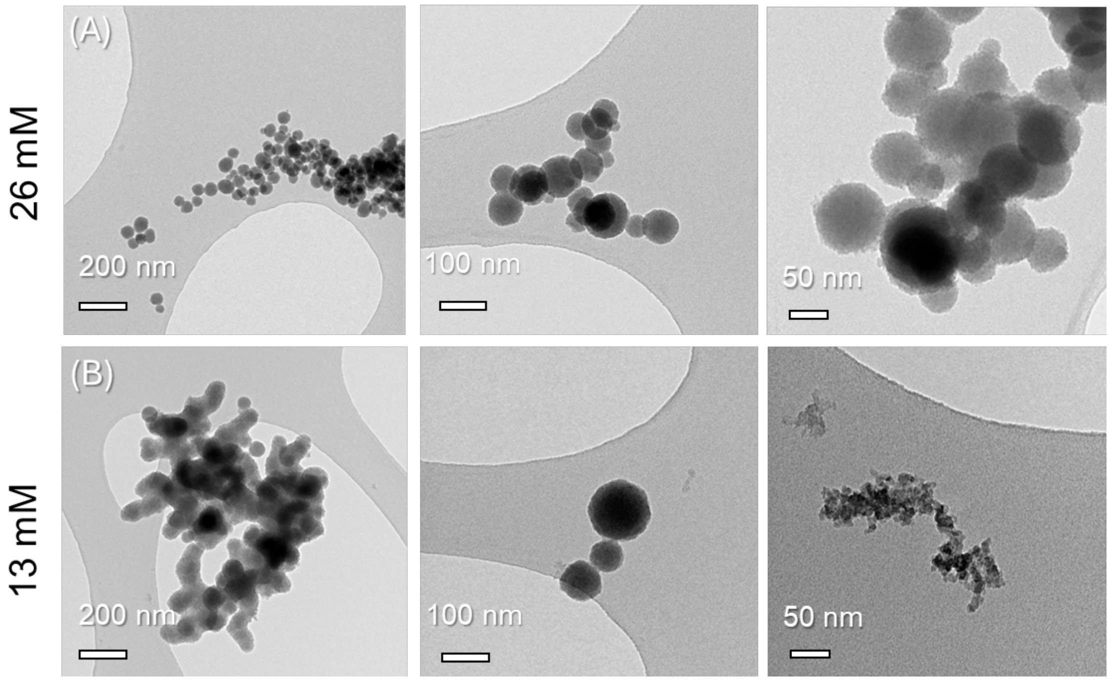

Figure S8. (A) TEM images of DFO@MSN-1 with DSDA concentration equal to 26 mM. (B) TEM images of DFO@MSN-3 reducing DSDA concentration to 13 mM.

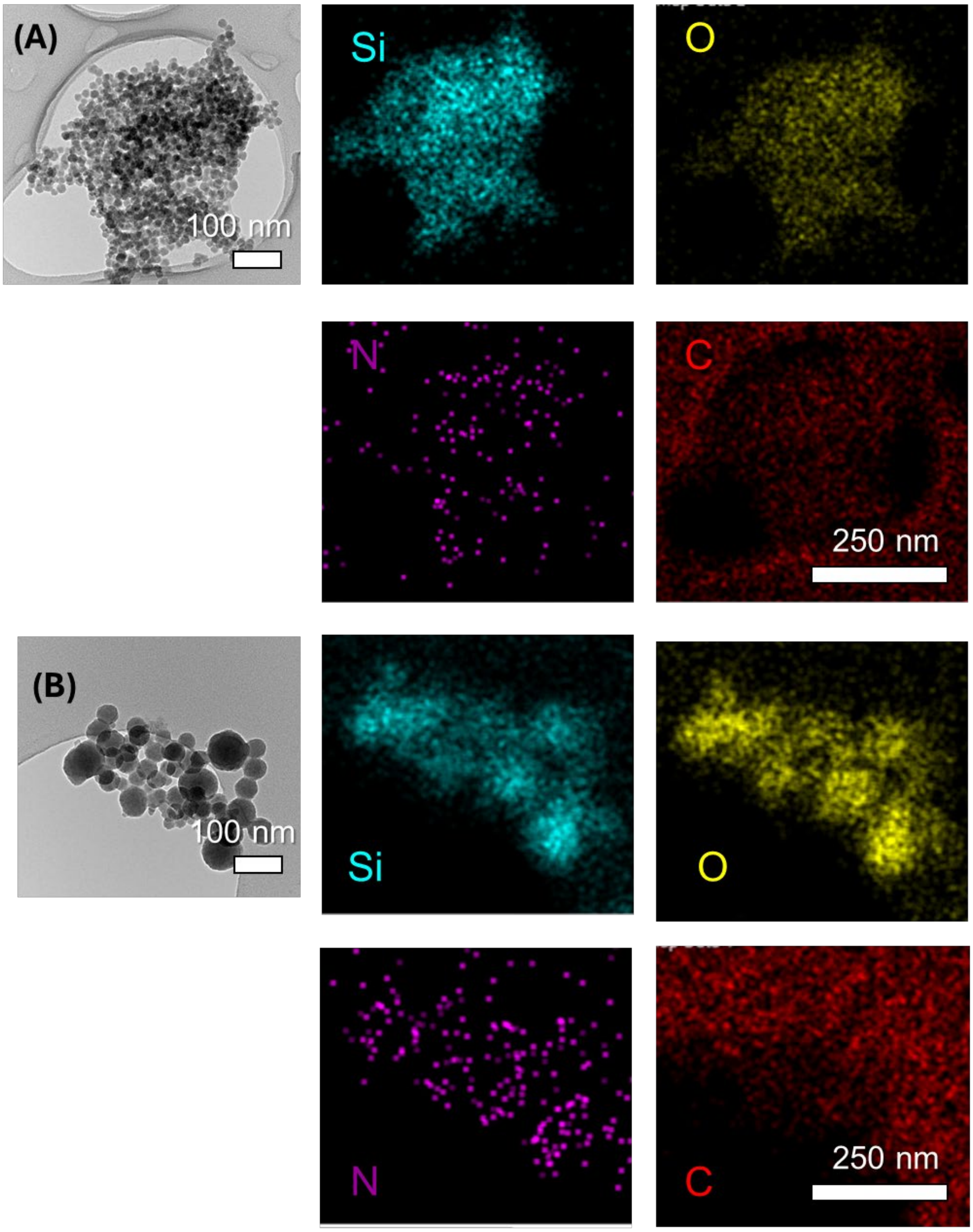

Figure S9. EDS mapping analysis for (A) DFO@MSN-20 and (B) DFO@MSN-40.

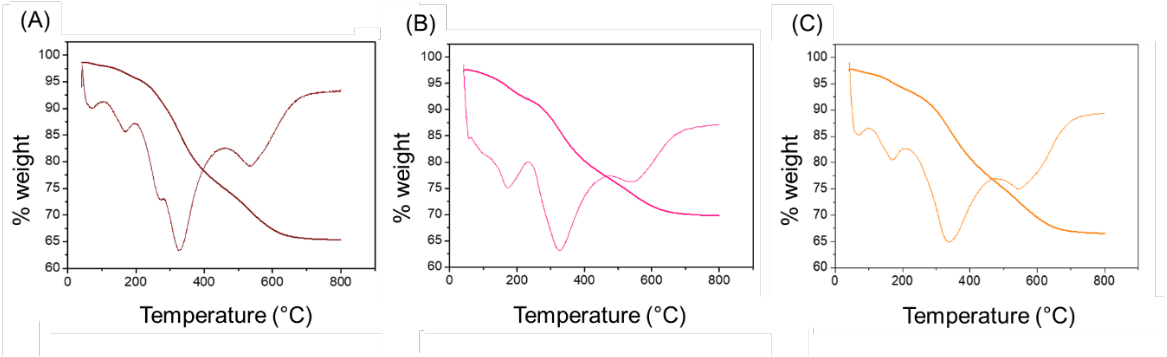

Figure S10. TGA and first derivative of weight loss of (A) DFO@MSN-20, (B) DFO@MSN-40 and (C) DFO@MSN-110.

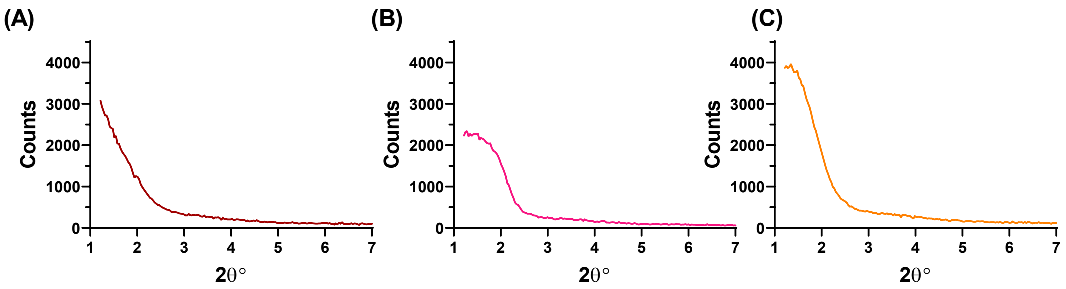

Figure S11. XRD patterns of (A) DFO@MSN-20, (B) DFO@MSN-40 and (C) DFO@MSN-110.

Table S4. Loading capacity of reference materials and DFO@MSN-40 determined by TGA.

	**mg DFO/ 100 mg MSN**	
	**Experiment A ($7\ mg{\cdot}mL^{-1}$)**	**Experiment B ($15\ mg{\cdot}mL^{-1}$)**
**MCM-41-NH_2**	4.2	1.5
**MCM-41-COOH**	6.8	6.9
**DFO@MSN-40**	10.1	

Table S5. Results of Kinetic modally of DFO release from DFO@MSNs and reference material at pH=7.4.

Nanomaterial	Higuchi $f(t)=K\,t^{1/2}$		Korsmeyer-Peppas $f(t)=K\,t^{n}$		
	K	R^2	n	K	R^2
**DFO@MSN-20**	0.0494	0.984	0.69	0.00007	0.956
**DFO@MSN-40**	0.0412	0.959	0.52	0.00077	0.972
**DFO@MSN-110**	0.0461	0.985	0.61	0.00020	0.977
**DFO@MCM-41-COOH**	0.1734	0.941	0.32	0.03639	0.898

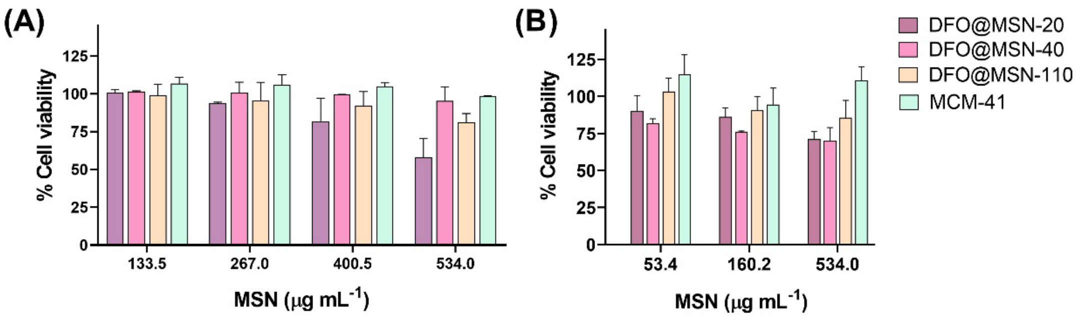

Figure S12. (A) Cell viability assays conducted in BV-2 at 72h in terms of nanoparticle concentration. MCM-41 was added as refence material. (B) Cell viability assays conducted in SH-SY5Y at 24h referenced to nanoparticle concentration.

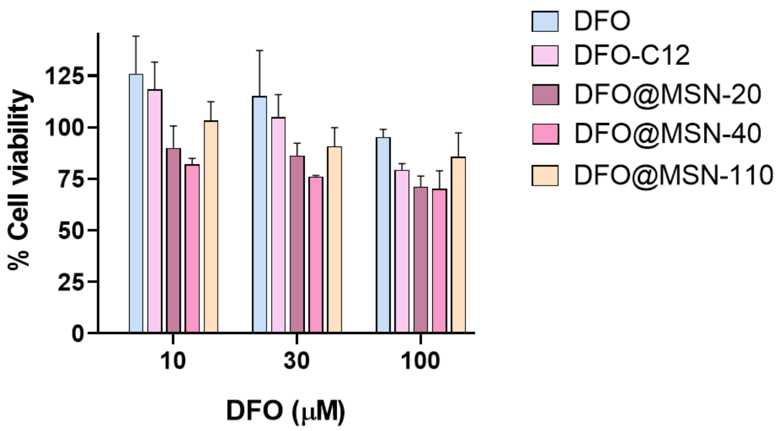

Figure S13. Cell viability assays conducted in SHSY-5Y at 24 h.

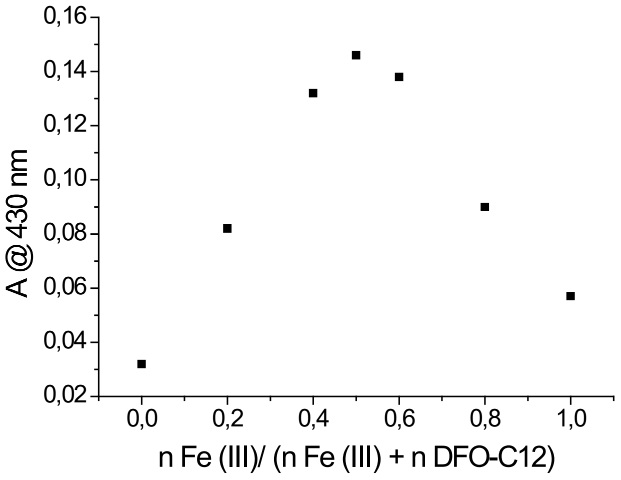

Figure S14. Job plot: determination of binding stoichiometry of DFO-C12 and Fe (III).

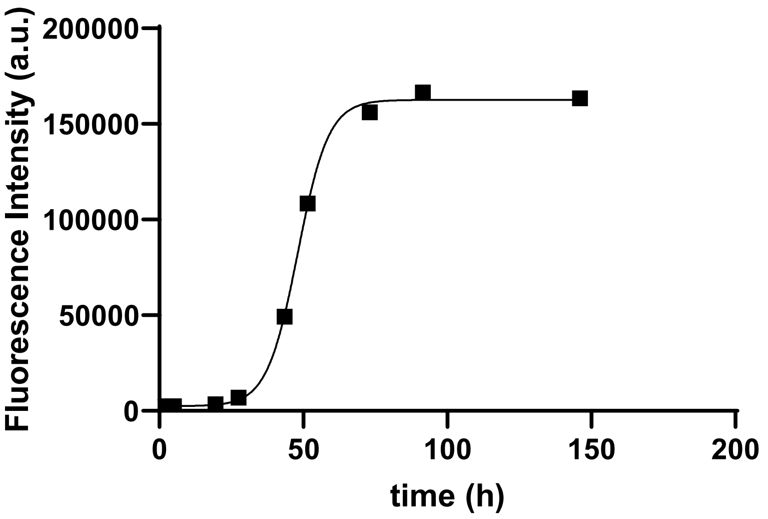

Figure S15. Kinetic folding of Lysozyme with Al (III).

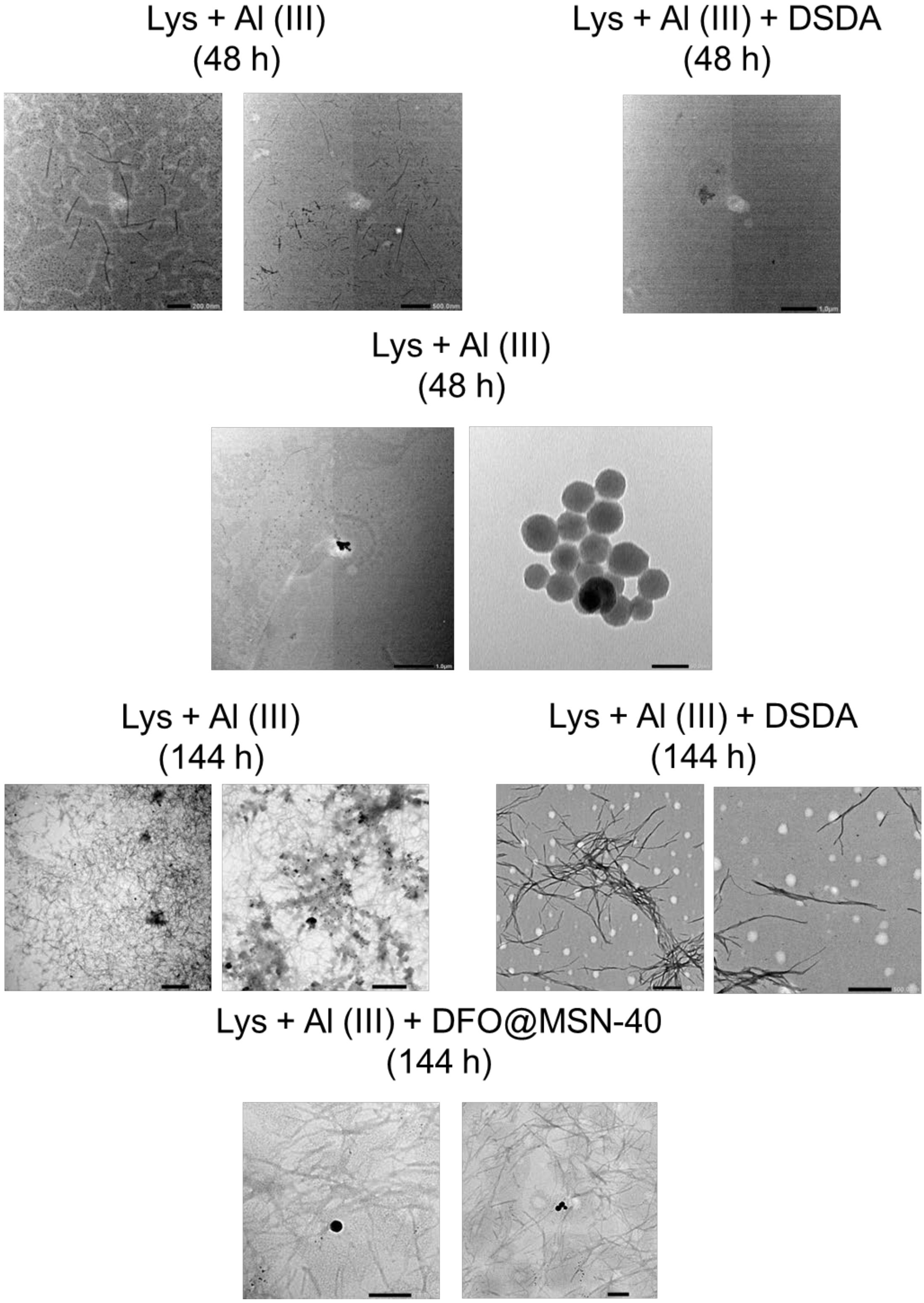

Figure S16. Negative staining TEM images of folding process of Lysozyme in presence of Al (III) and metal chelators.

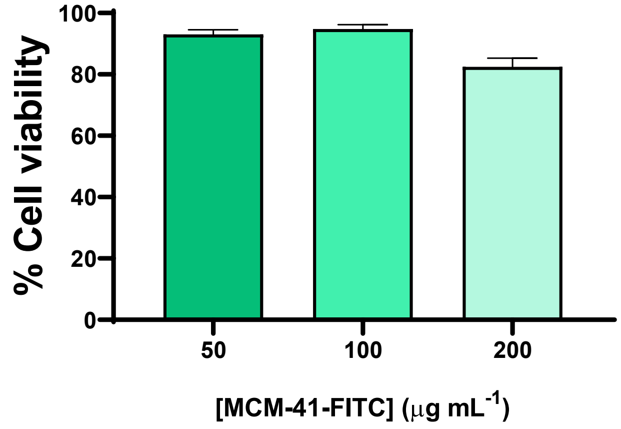

Figure S17. Cell viability using several concentrations of reference material in Caco-2 cell line at 72 h.
